# Simplification of biochemical models: a general approach based on the analysis of the impact of individual species and reactions on the systems dynamics

**DOI:** 10.1186/1752-0509-6-14

**Published:** 2012-03-05

**Authors:** Irina Surovtsova, Natalia Simus, Katrin Hübner, Sven Sahle, Ursula Kummer

**Affiliations:** 1 University of Heidelberg, Im Neuenheimer Feld 267, 69120 Heidelberg, Germany

## Abstract

**Background:**

Given the complex mechanisms underlying biochemical processes systems biology researchers tend to build ever increasing computational models. However, dealing with complex systems entails a variety of problems, e.g. difficult intuitive understanding, variety of time scales or non-identifiable parameters. Therefore, methods are needed that, at least semi-automatically, help to elucidate how the complexity of a model can be reduced such that important behavior is maintained and the predictive capacity of the model is increased. The results should be easily accessible and interpretable. In the best case such methods may also provide insight into fundamental biochemical mechanisms.

**Results:**

We have developed a strategy based on the Computational Singular Perturbation (CSP) method which can be used to perform a "biochemically-driven" model reduction of even large and complex kinetic ODE systems. We provide an implementation of the original CSP algorithm in COPASI (a COmplex PAthway SImulator) and applied the strategy to two example models of different degree of complexity - a simple one-enzyme system and a full-scale model of yeast glycolysis.

**Conclusion:**

The results show the usefulness of the method for model simplification purposes as well as for analyzing fundamental biochemical mechanisms. COPASI is freely available at http://www.copasi.org.

## 1 Background

Biochemical systems are inherently high dimensional due to the large number of interrelated cellular components and processes, the temporal organization of which spans time scales of several orders of magnitude. Aiming at a comprehensive understanding of the dynamic behavior of such systems has led to the development of an ever increasing number of computational models which are in the majority of cases formulated on the basis of ordinary differential equations (ODEs) [1]. Even though the purpose of computational models is to facilitate understanding and analysis of the underlying biochemical mechanisms, this again becomes cumbersome with the growing complexity of modern models. Therefore, it is important to identify those parts of the biochemical systems and of the model that are responsible for the observed physiological behavior. This necessitates the development of methods for the rational simplification of computational models and to make them comfortably accessible to the community.

Numerous methods have been developed to simplify (bio)chemical reaction networks (see review [2]). For biochemical systems many of the reduction methods aim at analyzing the steady state behavior either heuristically [3] or employing mathematical analysis (e.g. sensitivities [4,5]). Since biochemical systems usually do not reside in a steady state time-dependent approaches have recently been proposed (see for example [6,7]). Most of these use a mathematical analysis of the different time-scales occurring in the biochemical systems, e.g. the Intrinsic Low-Dimensional Manifolds (ILDM) method [8-11] and the Computational Singular Perturbation (CSP) method [12-14]. Apart from the advantage of a time-resolved analysis, these methods can provide useful insights, such as the support of the detection of fast reactions and species as well as the identification of potential rate controlling reactions. However, a disadvantage of the above methods is that the reduced models are systems of mathematically transformed differential or differential algebraic equations (DAE) which may not relate one-to-one to biochemical species and reactions hampering the biochemical interpretation. In contrast, the methods based on steady-state or partial equilibrium approximations keep the one-to-one relation and are therefore simple to biochemically interpret.

In this paper, we focus on deriving simplified biochemical models by discarding fast reactions and species. For this purpose we present a reduction strategy which is based on the CSP algorithm developed by Lam and Goussis [14]. The algorithm examines the time scales of ODE systems and supports the separation of the biochemical network into fast and slow subsystems. This is achieved through the elimination of the detected quasi-stationary species and quasi-equilibrium reactions.

The original CSP algorithm is implemented in the software COPASI [15] making it accessible to the scientific community. COPASI is a platform-independent, user-friendly software tool that allows easy access to powerful numerical methods for simulation and analysis of biochemical reaction networks.

We apply the simplification strategy to two different systems to exemplify its use. Thus, as a simple system, we present the derivation of the Michaelis-Menten Kinetics. As a realistic case, we analyze the glycolysis in *Saccharomyces cerevisiae *[16] in three different dynamic regimes. We show that several variables can be eliminated still keeping the original dynamics intact. Furthermore, regulatory mechanisms cause different players to participate with different relative importance in the dynamics of the system.

### Time Scale Separation Analysis

In order to explain the basic notions of a time scale decomposition we start with a first-order kinetics system. Then, the differential equations describing the system dynamics **y **are linear:

$$\frac{d\;y}{d\;t}=J\cdot y$$

with constant and diagonalisable Jacobian **J**. By using the set of right eigenvectors **A **of **J **as the new basis we can decompose the Jacobian [17] and transform the original equations into:

$$x=A^{-1}\cdot y,\;\Lambda =A^{-1}\cdot J\cdot A.$$

The components of the transformed concentration vector **x **are called *modes*. Because **Λ **is a diagonal matrix of real or complex eigenvalues *λ_i _*of **J**, the transformed ODE system is fully decoupled:

$$\frac{d\;x^{i}}{d\;t}=\lambda^{i}\;x^{i},\;i=1,\;\ldots ,\;N.$$

Thus, the modes $x^{i}\sim e^{\lambda^{i}t}$ evolve independently of each other. The reciprocals of ℜ(*λ^i^*):

$$\tau_{i}=-\frac{1}{ℜ\left(\lambda^{i}\right)}$$

have a dimension of time and are called time scales (TS). Ordering them w.r.t. magnitudes *τ*_1 _<*τ*_2 _< ... <*τ_N _*leads to approximate speed ranking of the modes [14]. The modes corresponding to fast time scales (eigenvalues with large negative real part) approach 0 very quickly and can be eliminated from the system for *t *≫ *τ_M _*, where *τ_M _*is a fast time scale.

Two additional aspects are worth being emphasized here. First, although the transformed representation of the system dynamics in terms of modes provides a systematic basis for the decomposition of the reaction network, it does not guarantee reducing the number of biochemical species or reactions in the system, since many different species might contribute to one and the same transformed equation. So, there is no straightforward relation to reduction methods commonly used in biochemistry such as the quasi steady state (QSS) approximation or the quasi equilibrium (QE) assumption.

An additional aspect of TS decomposition is that it is based on the local analysis of the system dynamics. For general nonlinear problems however the Jacobian is time-dependent. Its eigenvalues and eigenvectors change with time. Hence, in order to obtain a reasonable characterization of the systems dynamics the time scale decomposition has to be applied repeatedly at many time points through the evaluation time of the reaction system.

## 2 Results

### 2.1 CSP in COPASI

Consider a system consisting of *K *biochemical reactions, the dynamics of which is determined by a system of *N *ordinary differential equations:

(1) $$\frac{d\;y\;\left(t\right)}{d\;t}=g\left(y\left(t\right)\right)=\overset{R}{\underset{r=1}{\sum }}s_{r}F^{r}\left(y\right)$$

here **y **is the *N*-dimensional concentrations vector, **s_r_**(*r *= 1, . . . , *R*) are the *N*-dimensional stoichiometric vectors and *F^r^*(**y**) is the rate of the *r*-th reaction.

**The main idea of the CSP method **is to split the *N*-dimensional space of the vector **g **into two subspaces, a fast and a slow subspace:

$$g=g^{\text{fast}}+g^{\text{slow}}.$$

In general, an *N*-dimensional vector may be expressed in terms of any set of *N *linearly independent basis vectors (e.g. [17]). The objective of the CSP method is to express **g **in a new basis, one that is tuned to the dynamics of the system, where the fast and slow components evolve independently of each other.

The subspace **g**^fast ^relates to the fast time scales of the system. If its contribution is negligible (according to some error criteria), the original system (Eq. 1) simplifies to the system of the following differential algebraic equations (DAEs):

(2) $$\begin{array}{c} \frac{dy}{dt}\;\approx \;g^{\text{slow}},\;i=M+1,\ldots ,N,\\ g^{\text{fast}}\approx \;0,\;i=1,\ldots ,M. \end{array}$$

This DAE system does not contain the fastest time scales of the original model. Hence, it is much less stiff than the original system and can be simulated easily. Nevertheless such a simplification does not guarantee the reduction of the number of species and reactions ("real" system reduction) as explained above. Therefore, a similarly important aspect of our strategy is the identification of QSS metabolites and QE reactions by means of CSP data (see [14] and below).

#### The Algorithm

Let us differentiate equation (1) with respect to time and get the following form with the Jacobian **J**:

(3) $$\frac{d\;g}{d\;t}=J\cdot g\;\left(y\right),\;J=\frac{\partial g}{\partial y}.$$

Now we (again) focus on the choice of an appropriate basis **a***_i_, i *= 1, . . . , *N*, but in contrast to the approach of time scale separation, these linearly independent vectors do not have to be eigenvectors of the Jacobian **J **(not even orthogonal). Then **g **always has the unique representation:

$$g=\overset{N}{\underset{i=1}{\sum }}a_{i}\;f^{i}$$

where **a***_i _f^i ^*is a so-called reaction mode, the amplitude *f^i ^*is given by:

(4) $$\begin{array}{c} f^{i}\left(y\right)\equiv b^{i}⊙\;g=\overset{R}{\underset{r=1}{\sum }}B_{r}^{i}F^{r},\;i=1,2,\ldots ,N,\\ B_{r}^{i}\equiv b^{i}⊙\;s_{r},\;i=1,2,\;\ldots ,\;N,\;r=1,2,\;\ldots ,\;R. \end{array}$$

The notation ⊙ abbreviates the standard scalar product, i.e. here $b^{i}⊙a_{j}=\sum_{n=1}^{N}b_{n}^{i}a_{j}^{n}.$ The set of *N *row vectors **b***^i ^*are the inverses of **a***_i_*; together they satisfy the following orthonormal condition: $b_{i}⊙a_{j}=\delta_{j}^{i},\;i,j=1,2,...,N.$

The CSP provides an algorithm to determine the number of fast modes *M*, and to compute the sets of linearly independent **a***_i _*and **b***^i^*, such that

$$g=\overset{M}{\underset{i=1}{\sum }}a_{i}f^{i}+\overset{N-M}{\underset{i=1}{\sum }}a_{i}f^{i}.$$

Differentiating equation (4) with respect to time we get:

(5) $$\frac{d\;f}{dt}=\Lambda \cdot f,\;f=B\cdot g,$$

(6) $$\Lambda =\left(\frac{d\;B}{d\;t}+B\cdot J\right)\;\cdot A,\;A=B^{-1}$$

where **A **and **B **are matrices consisting of the column vector **a***_i _*and row vector **b***^i ^*as basis vectors.

For linear problems the matrix **Λ **is time independent. In this case the choice of eigenvectors of the Jacobian as the new basis leads to the diagonal matrix **Λ **(see above). The corresponding amplitudes **f **evolve independently of each other with their own characteristic time scale *τ_i_*. For general nonlinear systems, however, **Λ **is time dependent and usually not diagonal. The CSP method provides an iterative procedure of refinement of basis vectors **a***_i _*and **b***_j_*. When recursively applied, the refinement procedure weakens the coupling between the *M *fast and the *N - M *slow amplitudes. The matrix **Λ **built from the final refined set of basis vectors is block-diagonal and the fast amplitudes are uncoupled from the slow ones approximately, so that the residual coupling can be neglected.

The process starts with an arbitrary initial guess for the basis vectors **a***_i _*and the assumption that the first *M *basis vectors span the *M*-dimensional fast subdomain. The corresponding time scales should be much faster than some characteristic time scale of interest, then *M *is to be selected to provide a gap between the slow (time of interest) and fast time scales:

(7) $$\frac{\tau_{M}}{\tau_{M+1}}\ll \varepsilon .$$

When for the final set of basis vectors the sum of M fast reaction modes falls below some user-specified threshold:

(8) $$|\overset{M}{\underset{i=1}{\sum }}a_{i}f^{i}\tau_{M}|\;<y_{\text{error}}=\varepsilon_{\text{rel}}\cdot y_{j}+\varepsilon_{\text{abs}}$$

these can be eliminated from the initial system (Eq. 1), because their contributions to **g **are negligible. As a consequence, the evolution of the reduced system depends on the slow modes only (see Eq. 2).

#### Implementation

The CSP algorithm was implemented as an integral part of the COPASI software in C++ and is freely available with the current releases of the package. In this implementation, the CSP algorithm is applied to the models for which linear dependencies due to conservation relationship are eliminated. This is achieved by the analysis of the stoichiometric matrix and is performed by COPASI automatically.

The following **CSP parameters **have to be defined by the user.

##### Intervals

The user specifies the number of time points for which the CSP analysis is carried out by setting the time interval. The time interval should be large in comparison with the user's time scale of interest.

##### Ratio of time scale separation *ε*

This parameter specifies the gap between the time scales related to the fast and slow modes (Eq. 7).

##### Error tolerance

Absolute *ε*_abs _and relative error *ε*_rel _are set to control when a fast mode is considered to be exhausted (Eq. 8).

The CSP algorithm described above provides local information at certain time steps. To obtain global features of the system behavior the analysis must be performed at all points in the range of interest. For this purpose the CSP step involves numerical integration using the LSODA solver [18]. LSODA is part of the ODEPACK library [19]. It solves ODE systems with a dense or banded Jacobian when the problem is stiff, but it automatically selects between non-stiff (Adams) and stiff (BDF) methods. The Jacobian is generated numerically.

When the CSP algorithm at time point *t *has been performed and both final refined sets of basis vectors **a***_i_*(*t*) and **b***^i^*(*t*) are available the *M*(*t*) is set to the number of fast exhausted modes and *τ_M_*(*t*) is then the time scale of the slowest of fast reaction modes at time point *t*.

The CSP output data (see below) can either be exported to a text file (save as Report in COPASI) for the use in other software (gnuplot, Octave etc.) or displayed in the graphical user interface as tables. In this case a color coding is used where the numbers are additionally visualized by different shades of color. This makes it easy to immediately spot e.g. the most important contributions to a specific mode for a large model (where the result tables are correspondingly large).

**Figure 1:**
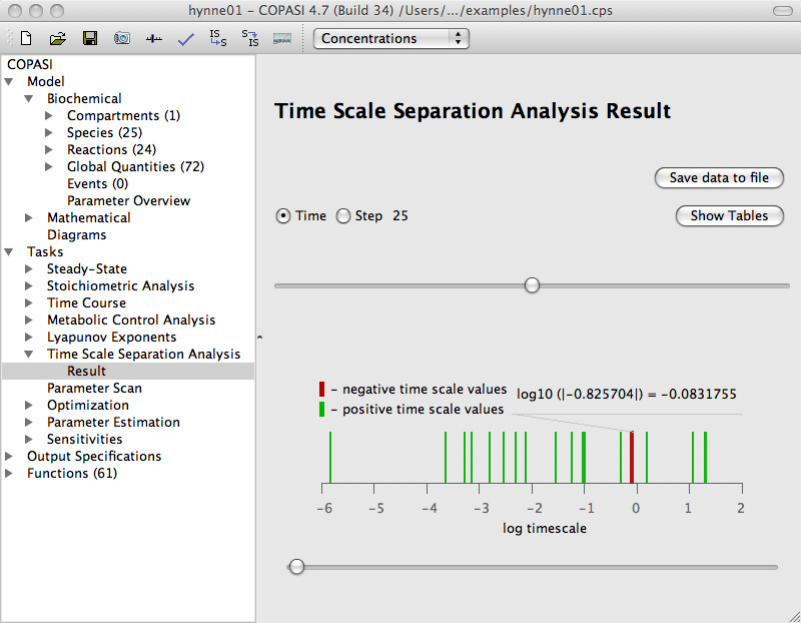
**COPASI visualization of the time scale distribution**. Full glycolysis model of Hynne et al. [16], [Glc*_x_*]_0 _= 14 mM, *t *= 25 min. The coincident bars on the graph correspond to equal time scales.

We also use three dimensional bar graphs for visualizing the matrices employing the qwtplot3D library (http://qwtplot3d.sourceforge.net) integrated in COPASI. These bar graphs can be turned and zoomed interactively. Furthermore single rows or columns of the matrix can be highlighted. An additional diagram shows the distribution of the time-scales of the different modes at chosen points of time (Figure 1). Applying the time slider in the graphical user interface it is very simple to switch between the results for different time points. Therefore the user can easily get an overview of the time-dependent changes of the time-scale separation.

#### CSP Data used for model analysis

The CSP algorithm supplies the modeler with local CSP output data [14] that relates the time scales to species and reactions of the original biochemical system. The data is computed by the help of the refined sets of basis vectors **a***_i_*(*t*) and **b***^i^*(*t*). The user is provided with the CSP output at each defined time point during the interval of interest and can use it to reduce the model in a rational way. The CSP output data are displayed in COPASI in a number of matrices. Here we briefly explain the most important CSP output data which are available in COPASI:

##### Time scales

The analysis of time scales evolution can provide useful information about the system dynamics. The fast dissipative time scales relate to the eigenvalues of the Jacobian with large negative real parts. The explosive modes are associated with positive eigenvalues. Modes with equal time scales correspond to pairs of complex conjugate eigenvalues indicating oscillatory components in the system behavior.

##### Radical Pointer (RP)

The CSP Radical pointer identifies the species for which the QSSA can be justified. Whenever the *i*-th diagonal element of *m*-th fast mode projection matrix *Q_m _*= **a***_m_***b***^m ^*is not a small number, species *i *is said to be a CSP radical.

##### Participation Index (PI) and QE reactions

The relative level of participation of the *r*-th elementary reaction to the n-th CSP reaction mode can be represented by the mode participation index, $P_{r}^{i}$, defined as follows:

$$P_{r}^{i}\equiv \frac{B_{r}^{i}F^{r}}{\overset{R}{\underset{r=1}{\sum }}{|B}_{r}^{i}F^{r}|+|\frac{b^{i}⊙y_{\text{error}}}{\tau_{M+1}}|},$$

where *i *= 1, . . . , *N, r *= 1, . . . , *R*.

##### Importance Index (II)

The relative importance of the contribution of the *r*-th elementary reaction to the rate of change of the *i*-th element of **y **can be represented by the importance index, $I_{r}^{i}$:

$$I_{r}^{i}\equiv \frac{s_{r}^{i,\text{s}1\text{ow}}F^{r}}{\overset{R}{\underset{r=1}{\sum }}|s_{r}^{i,\text{s}1\text{ow}}F^{r}|+\left|\frac{y_{\text{error}}}{\tau_{M+1}}\right|},$$

*i *= 1, . . . , *N, r *= 1, . . . , *R*. An effective stoichiometric vector of the *r*-th elementary reaction, $r_{r}^{\text{slow}}=\left(I-Q\left(M\right)\right)⊙s_{r}$ is computed using the fast subspace projection matrix $Q\left(M\right)=\overset{M}{\underset{m=1}{\sum }}Q_{m}$. The reaction with the largest $I_{r}^{i}$ for the species *y_i _*is the rate controlling reaction.

#### CSP - based model reduction

In this paragraph we summarize the most important steps in the reduction of the kinetic mechanism based on the results of the CSP algorithm described above. Model reduction is mainly the outcome of a sequence of QSSA for species and QEA for reactions which leads to the lumping or elimination of corresponding variables. The QSSA identifies species whose production and destruction rate are in approximate balance. Mathematically it means that the right-hand side of the corresponding differential equation is zero. The QE assumption corresponds to reactions whose forward and reverse rates are nearly equal (see for instance, [20]). In either case an approximate algebraic relation (equation of state) is obtained between participating species.

As described in the previous paragraph the CSP method provides the numerical data (RP, PI and II) that are an effective diagnostic tool allowing the detection of species which can be approximated by an equation of state, as well as the determination of the relative level of participation of distinct reactions to the modes.

In contrast to the original CSP method [14] we introduce and use the "subspace" radical pointers and the "subspace" participation indices rather than the individual mode RP and PI. This is based on the fact, that even though the matrix **Λ **(Eq. 6) built by the help of the final refined set of the basis vectors is block-diagonal and the fast modes are decoupled from the slow ones, the fast and slow modes could be coupled between themselves. So, it appears to be more reasonable to consider a projection of the CSP indices on the full fast and slow subspaces.

We consider the sum of all CSP radicals as selected by *M *fast modes and define the species with the largest "fast subspace" radical pointers as QSS. Similarly the sum of Participation indices over all slow and fast modes should be considered separately in order to detect the fast reactions. The normed PIs over fast and slow subspaces are:

(9) $$PI_{k}^{\text{fast}}=\frac{\overset{M}{\underset{i=1}{\sum }}PI_{k}^{i}}{\overset{N}{\underset{i=1}{\sum }}PI_{k}^{i}},\;PI_{k}^{\text{slow}}=\frac{\overset{N}{\underset{i=M+1}{\sum }}PI_{k}^{i}}{\overset{N}{\underset{i=1}{\sum }}PI_{k}^{i}},$$

We declare the reaction *k *as QE, if it is active in the fast and does not influence the slow space: $PI_{k}^{\text{fast}}\gg PI_{k}^{\text{slow}}$ at all time points, where the CSP analysis was carried out.

Practically, there exist only very few guidelines in the literature for deriving model simplifications based on the QEA and QSSA. Therefore, we would like to quickly summarize the procedure for the CSP-based model simplification:

1. First, a time scale of interest should be selected. This can for instance correspond to the time resolution of the experiment which is the basis for the model. The aim of the model simplification is to reduce all scales that are faster than this chosen scale.

2. Second, user defined parameters have to be selected in COPASI as explained above. Since the CSP information will be available for every time interval and is the basis for the time-dependent model reduction, the time interval should be large enough in comparison with the time scale of interest.

3. Third, performing the CSP and analyzing the results in order to find the QSS species and QE reactions.

4. Fourth, solving the corresponding algebraic equations of state and eliminating the respective variables from the reaction networks. The kinetic laws for slow reactions should be modified by substitution with explicit expressions for CSP radicals.

5. Fifth, parameter adjustment (e.g. by parameter estimation) with respect to quasi equilibrium constants of eliminated reactions in order to achieve the desired accuracy.

It is worth mentioning that during the simplification the existing conservation laws have to be preserved. The algebraic equations should be solved under conditions that the equations of moieties are fulfilled.

### 2.2 Application examples

We have applied the method to two models. All informations and scripts needed to reproduce the figures in this subsection are available in Additional Files 1, 2, 3.

#### Michaelis-Menten Kinetics

As in [10] we start our discussion with the simplest enzymatic reaction mechanism, the irreversible Michaelis-Menten kinetics:

SSubstrate+ EEnzyme ⇔k-1k1 CComplex→k2 PProduct+ E.Enzyme

The model was build in COPASI and consists of the two reactions R_1 _(*S *+*E *⇔ *C*) and R2 (*C *→ *P *+*E*). In order to illustrate the handling of the CSP based model reduction we consider two limit situations for the dimensionless parameters: $S_{t}=\frac{k_{2}}{k_{-1}}\to 0$ and $M_{r}=\frac{E_{0}}{S_{0}}\to 0$ (here *E*_0 _and *S*_0 _are initial enzyme and substrate concentrations, respectively). We used the following CSP parameter values: *ε *= 0.01, *ε*_rel _= 10^-5^, and *ε*_abs _= 10^-10^. In both cases, a clear time-scale separation occurs.

(**i**) **M_r _→ 0 **(**E_0 _≪ S_0_**)*: *This is the standard situation for Michaelis-Menten kinetics. The motion on the fast time-scale is dominated by the complex *C *decoupled from the substrate *S*. On the slow part, the changes of substrate and complex are balanced. The quasi steady state assumption for complex *C *leads then to the Michaelis-Menten kinetic law.

**Figure 2:**
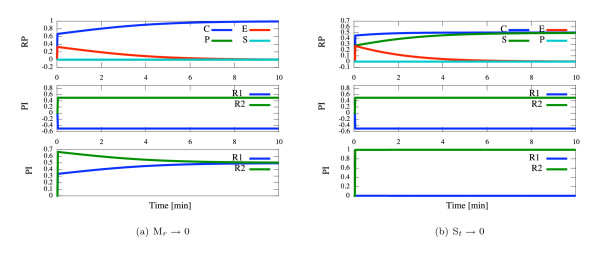
**Michaelis Menten model**. Left: *M_r _*→ 0 (*S*_0 _= 100; *E*_0 _= 1; *k*_1 _= 1; *k*_-1 _= *k*_2 _= 100). Right: *S_t _*→ 0 (*S*_0 _= 100; *E*_0 _= 100; *k*_1 _= *k*_*-*1 _= 100; *k*_2 _= 1). Time evolution of the Radical Pointer (RP) in the fast mode (top), Participation Indexes (PI) of reactions *R*_1 _and *R*_2 _in the fast (middle) and slow mode (bottom). The RP of product P in the first case *M_r _*→ 0 is similar to RP of substrate S (the both lines overlaid).

The CSP method allows the distinction between slow and fast modes (for times *t >*0.03). The Radical Pointer from the CSP data shows that the complex *C *dominates the fast mode. The contributions of both reactions to the slow and fast modes are comparable (see Figure 2, which displays the evolution in time of Radical Pointer and Participation Indices). Thus, the QSSA for the complex *C *is justified in this case.

(**ii**) **S_t _→ 0 **(**k_2 _≪ k_-1_**). This limit means that an equilibrium between the enzyme *E*, the substrate *S *and the enzyme-substrate complex *C *is established quickly. The slow step is the breakdown of *C *to produce the product *P *and the enzyme *E*.

The CSP analysis leads to the occurrence of two independent dynamical modes. After the short transient phase (*t <*0.006, when no reduction is possible) the contribution of *C *to the fast mode is larger than the one of *S *(nevertheless no real dominance occurs). Over the time the contribution of both variables becomes equal (Figure 2). Thus, the QSSA for complex *C *is incorrect.

Nevertheless, there is a clear separation of reactions in the modes. The reaction *R*_2 _of product formation dominates clearly the slow mode. Both reactions are active in the fast mode (see Figure 2). Thus, the reaction $R_{1}:S+E\overset{k_{1}}{\underset{k_{-1}}{\Leftrightarrow }}C$ is always practically in equilibrium and the QEA for reaction *R*_1 _is correct and leads to a similar equations as for "standard" Michaelis-Menten kinetics (compare [21] and [10]):

$$\frac{dP}{dt}=\frac{k_{2}E_{0}S}{K_{s}+S},\;\;\text{with}\;\text{equilibrium}\;\text{constant}\;K_{s}=\frac{k_{-1}}{k_{1}}.$$

The reader is referred to Additional File 1 for more details of the reduction of the Michaelis-Menten kinetics.

#### Glycolysis in *Saccharomyces cerevisiae*

We now use the CSP method to examine a more complex model for simplification purposes. We take the quantitative model of yeast glycolysis developed by [16] as an application example which has been also used before in similar studies [13,22].

**Figure 3:**
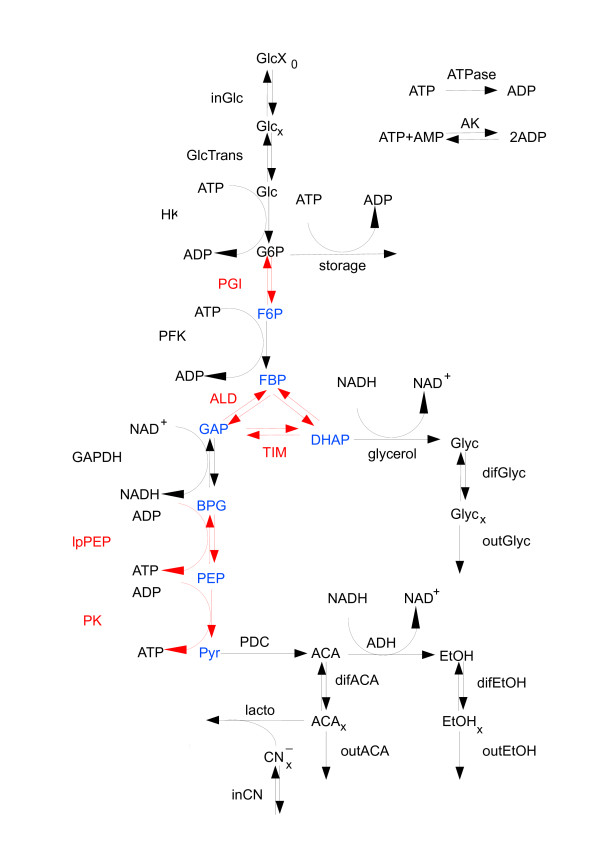
**Reaction scheme for the glycolysis model of *S. cerevisiae***. Fast reactions are marked in red, reduced (or lumped) metabolites in blue.

The model is based on ODEs and consists of 24 reactions among 22 metabolites with a total of 59 kinetic parameters. The reaction scheme is depicted in Figure 3. From the reaction stoichiometries two moiety conservations are derived:

$$\begin{array}{cc} \text{NAD}\;\text{ + }\;\text{NADH = const;}\\ \text{ATP + ADP + AMP}\;\text{ = const}\text{.} & \end{array}$$

The complete model is available for download in SBML format at the BioModels database [23] (BIOMD 61) or JWS online [24] (http://jjj.biochem.sun.ac.za/database/hynne/Hynne.xml), the latter version being used in this study. For model details the reader is also referred to [16]. However, there are some model properties we want to mention here explicitly.

The model reproduces experimental data observed in intact yeast cells in a continuous-flow stirred tank reactor. Here, the mixed flow glucose concentration, [Glc*_x_*]_0_, is a bifurcation parameter which means that depending on its value the system behavior changes qualitatively. To be concrete, this glycolysis model exhibits two stationary (*<*9.6 mM; 16.7 *<*[Glc*_x_*]_0 _*<*18.5 mM) and two oscillatory state regimes (9.6 ≤ [Glc*_x_*]_0 _≤ 16.7; ≥ 18.5 mM). Please refer to Figure eight in [16] for the bifurcation diagram. Notably, the first oscillatory regime has not yet been observed in experiments. So, we consider this as an important model property.

##### First step: CSP Analysis in COPASI

When performing a model reduction analysis it is indispensable to determine beforehand which properties of the system are to be maintained in the simplified model. We aimed at preserving (within an acceptable error range) the following features in order of priority:

1. A Hopf bifurcation occurs at some value of [Glc*_x_*]_0_.

2. Bifurcation points w.r.t. [Glc*_x_*]_0 _change only little, i.e. different dynamic regimes (including the first oscillatory domain) appear at values of [Glc*_x_*]_0 _close to the corresponding values in the full system.

3. Steady state levels of metabolite concentrations.

4. Periods of the oscillations.

5. Amplitudes of the oscillations.

We, therefore, perform the CSP analysis on the different dynamic regimes separately, i.e. using three different initial conditions for [Glc*_x_*]_0_, namely 9 mM (steady state), 14 mM and 24 mM (first and second oscillatory state, respectively). All other parameters of the model are taken as in [16].

**Figure 4:**
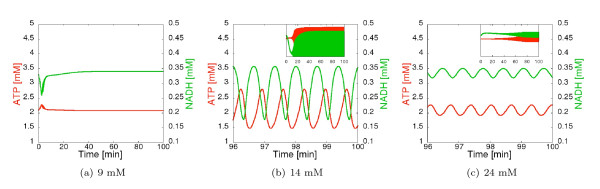
**Simulated time courses of [ATP] and [NADH] in the three different dynamic regimes at concentrations of [Glc*_x_*]_0 _from time *t *= 0 min to *t *= 100 min**. In Figure (b), (c) the subinterval from *t *= 96 min is drawn to a larger scale.

For each CSP analysis we simulate the system for a time period from 0 to 100 min, thereby taking also the initial transients into consideration, and inspect 250 time points along the trajectory which yields a time interval of 0.4 min. At each time point a full set of CSP data is computed. Example time course trajectories of the concentrations of ATP and NADH are shown in Figure 4. The CSP parameters *Ratio of mode separation, Relative Error *and *Absolute Error *are set to 0.99, 1e-3 and 1e-4, respectively.

In the following, we present the CSP output data (Time Scales, Radical Pointer, Participation Index, Importance Index and so on, see 2.1) one after the other. For each type of data, we point out the major differences between the three dynamic regimes which we interpret as glucose-dependent phenomena. If appropriate, special emphasis is given to time-dependent differences.

Since the amount of data produced in this comprehensive analysis exceeds the scope of the paper we present each CSP output data with compelling examples. The complete set of data is provided in Additional file 2.

###### Time scales

**Figure 5:**
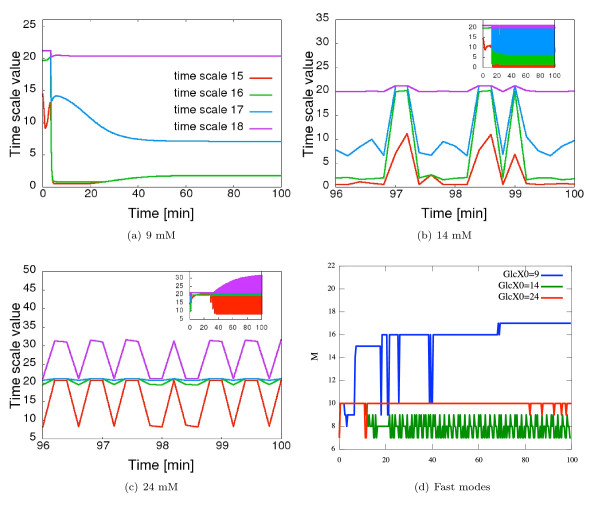
**Time evolution of the time scales 15 to 18 in the three dynamic regimes (a)-(c) at concentrations of [Glc*_x_*]_0 _from time *t *= 0 min to *t *= 100 min**. In Figure (b), (c) the subinterval from *t *= 96 min is drawn to a larger scale. (d) Time evolution of the number of fast modes *M *in the three different dynamic regimes.

The full model exhibits in total 20 different time scales with values that span about seven orders of magnitude (from *min *to *ms*). Figure 1 shows the time scale distribution (logarithmic values) of the full model exemplarily for [Glc*_x_*]_0 _= 14 mM at time step 25. Notably, the time scale values change over time. In the steady state regime ([Glc*_x_*]_0 _= 9 mM), we observe two eigenvalue pairs corresponding to the 8*^th ^*and 9*^th ^*as well as 15*^th ^*and 16*^th ^*time scales that consist of complex conjugates (*τ*_8 _= *τ*_9_, *τ*_15 _= *τ*_16_) indicating the system's intrinsic oscillatory vicinity. We see that the real part of these eigenvalue pairs become equal at a certain point in time during the initial transient (Figure 5(a)).

In both oscillatory state regimes, after the initial transients, the values of time scales become oscillating and show in part substantial amplitudes which sometimes also overlap with the values of adjacent time scales. As an example, Figures 5(b) and 5(c) show the time evolution of the 15*^th ^*to 18*^th ^*time scales for [Glc*_x_*]_0 _= 14 and 24 mM, respectively.

*Number of fast modes M *(Figure 5(d)): As explained above, each time scale corresponds either to a fast or slow so-called mode in the CSP analysis. Like the values of the time scales the number of modes constituting the entire fast or slow subspace changes over time. Since for model reduction only the fast modes are relevant we focus on these. Initially, all three dynamic regimes show seven fast modes. In the steady state regime, after a highly variable transient, *M *settles to 17. In contrast, *M *varies between 7 and 9 for the first and between 9 and 10 for the second oscillatory regime. Consequentially, we do not fix the number of fast modes in our CSP analysis but rather take their varying number over time into account in search for QSS metabolites (see RP) and QE reactions (see PI).

###### CSP Radical Pointer

**Figure 6:**
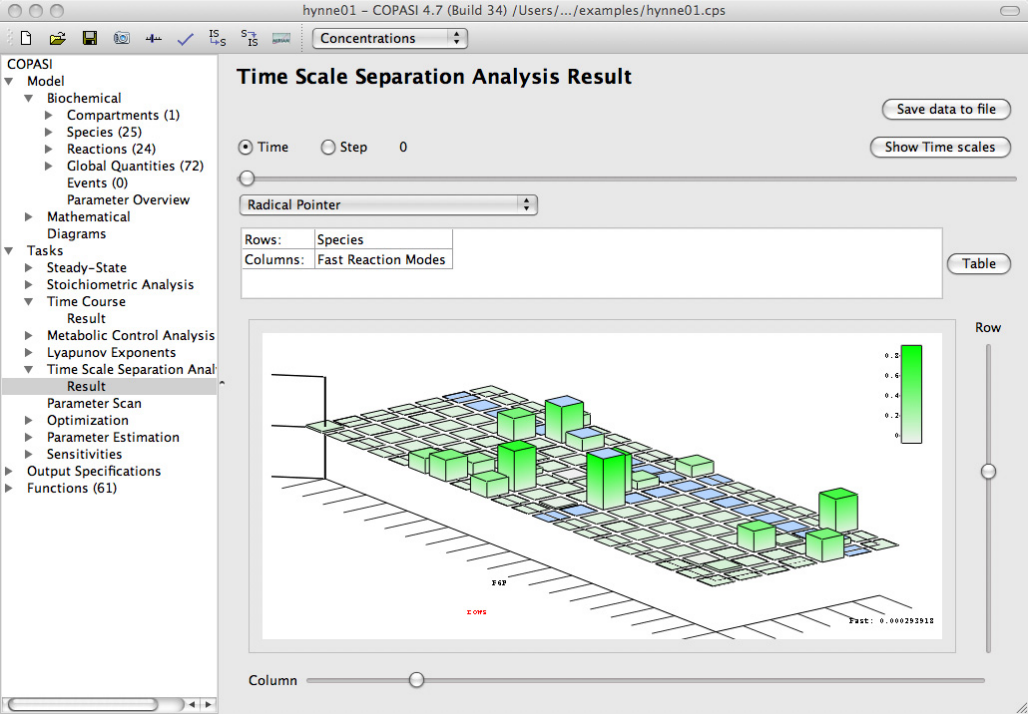
**COPASI bar graph visualization of the Radical Pointers**. Full glycolysis model of [16], [*Glc_x_*]_0 _= 24 at time *t *= 0. The 3D columns display the values of Radical Pointer as bars. One bar corresponds to one species (row) and one fast reaction mode (column).

Figure 6 shows how Radical Pointers are visualized in COPASI. Five metabolites (BPG, GAP, PEP, F6P and NAD) are fast in all of the three dynamic regimes.

###### CSP Participation Index (PI)

When comparing the normed sum of PIs for the three different regimes, four different categories of reactions can be identified depending on their respective PIs, e.g. the reaction can always be classified as fast or it changes its role between regimes. A heuristic threshold value based on our analysis and experience is chosen. Thus, if the normed sum of PIs over all fast modes exceeds 0.7, the reaction is defined as fast.

1. *"fast - fast - fast"*: vGAPDH, vlpPEP, vPK, vPGI, vALD, vTIM and vAK are fast in all regimes. These reactions, therefore, may be approximated as QE and eliminated in a simplified model. Not surprisingly, the known fast reactions vPGI and vTIM turn up in this group. Interestingly, the group also contains all reactions that either produce energy or redox equivalents, i.e. ATP and NADH, respectively. Obviously, especially in case of reactions being at the edge of the threshold, model reduction still has to be done with care.

2. *"fast - slow - slow"*: vHK, vPFK, vPDC, glycerol production, glycogen production, and ATP consumption are reactions that belong to this group which switch from fast to slow with increasing [Glc*_x_*]_0_. These reactions (except vPDC) share the property of consuming energy and redox equivalents, i.e. ATP and NADH, respectively. The continuous flow transport reactions between the outside and the chemostat (vinCN, vinGlc) as well as vlacto also belong to this group.

3. *"fast - slow - fast"*: vADH and the transport reactions across the cell membrane (vGlcTrans, vdifACA, vdifEtOH, vdifGlyc) behave differently from all others as they are fast for low and high concentrations of [Glc*_x_*]_0_. Participation in slow modes seems to be limited to the first oscillatory regime.

4. *"slow - slow - slow"*: All reactions from the chemostat to the outside (voutEtOH, voutGlyc, voutACA) are slow in all regimes.

**Figure 7:**
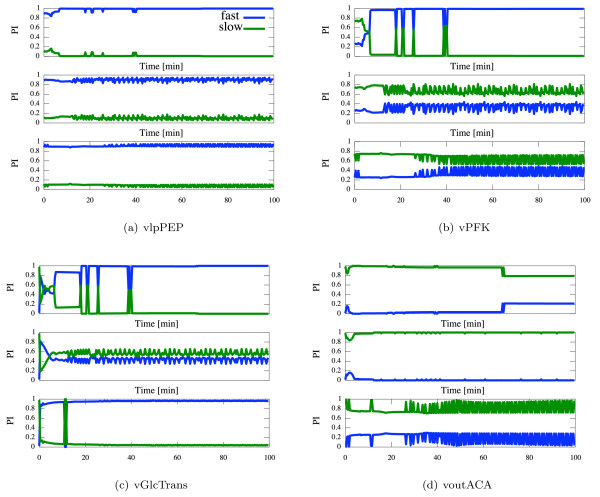
**The time evolution of the normed sum of Participation Indexes (PI) for vlpPEP (a), vPFK (b), vGlcTrans (c) and voutACA (d)**. Upper, middle and lower panel relate to [Glc*_x_*]_0 _= 9 mM, 14 mM and 24 mM, respectively. Blue and green curves show the contribution to the entire fast and slow subspace, respectively.

A typical example of time evolution of the normed PIs for each class of reactions is given in Figure 7.

###### CSP Importance Index (II)

The majority of reactions exhibit significant importance on a number of metabolites (Normed Importance Index *>*0.1). Exceptions are vPGI, vALD, vTIM, vlpPEP, vPK, vconsum, vAK and vdifACA, where Importance Indices are of values less than 0.1 for all metabolites. The weak importance of the first five reactions (already indicated as QE by the normed PIs) further confirms that they may be removed from the model. In some cases, the importance index changes in between regimes, depending on [Glc*_x_*]_0_. Examples for glucose-sensitive importance are vinGlc (important at low and unimportant at high glucose concentrations), vHK, vPFK and vGAPDH (unimportant at low and important at high glucose concentration). Obviously, the importance index gives similar information as control coefficients derived from MCA, a fact that we studied and verified (data not shown). However, the CSP IIs give a richer picture of the control distribution compared to MCA.

##### Second Step: Model Reduction

Based on the time scale separation analysis we suggest four steps to derive a simplified minimal model. A short description is given in the following. For any detail the reader is referred to Additional file 3. Each simplification step concerns a subset of the original model scheme which we call Module, hereafter.

*Module 1*. **QEA for vPGI, AE for F6P**. The normed PI revealed that PGI can be approximated as QE and the Radical Pointer of the 5-th fast mode identifies F6P as CSP Radical, for which the algebraic equation holds

$$K_{\text{PGI}}\approx \frac{\text{F}6\text{P}}{\text{G}6\text{P}}.$$

So, in order to eliminate F6P from the system and to lump PGI together with PFK we need to modify the chemical equation of the PFK reaction to

G6P+ATP→FBP+ADP

as well as the kinetic rate law to

$$\frac{V_{5m}\cdot {\left(K_{PGI}\cdot \;G6P\right)}^{2}}{\left(K_{5}\cdot \;{\left(1+\kappa_{5}\cdot \;\left(\frac{ATP}{AMP}\right)\right)}^{2}+{\left(K_{PGI}\cdot \;G6P\right)}^{2}\right)}.$$

*Module 2*. **QEA for vALD and vTIM**. The normed PI revealed that vALD and vTIM can be approximated as QE, for which the equations hold

$$K_{\text{ALD}}\approx \frac{\text{GAP}\cdot \text{DHAP}}{\text{FBP}}\;\;\text{and}\;\;K_{\text{TIM}}\approx \frac{\text{GAP}}{\text{DHAP}}.$$

The metabolites which are either substrate or product of the two reactions are FBP, DHAP and GAP. The latter is identified as CSP Radical (see Radical Pointer of the 2-nd fast mode). In order to lump vALD and vTIM together we introduce a pool metabolite which we name

trioseP=GAP+DHAP+FBP

and express any of the three metabolites in terms of *trioseP*. The new chemical equations of the associated reactions are:

$$\begin{array}{ccc} PFK & :\;\;G6P+ATP\to 2\cdot trioseP+ADP, & \\ GAPDH & :\;\;trioseP+NAD\to BPG+NADH,\\ Glycerol\;branch\;\; & :\;\;trioseP+NADH\to Glyc+NAD. \end{array}$$

*Module 3: ***QEA for vlpPEP**. The equilibria for the vlpPEP reaction is expressed as:

$$K_{\text{PEP}}\approx \frac{\text{BPG}\cdot \text{ADP}}{\text{PEP}\cdot \text{ATP}}.$$

BPG is identified as CSP Radical in the first mode and at the same time PEP in the 4-th mode. Again, we introduce a pool metabolite

BPG_PEP=BPG+PEP

and reduce the vlPEP reaction from the network. The new chemical equations of the associated reactions are:

$$\begin{array}{c} GAPDH\;\;:\;\;trioseP+NAD\to \text{BPG\_PEP}+NADH,\\ \;\;\;\;PK\;\;\;:\;\;\text{BPG\_PEP}+2ADP\to Pyr+2ATP. \end{array}$$

*Module 4: ***QEA for vPK**. The vPK reaction is modeled as irreversible. So, the QEA leads to its lumping together with vPDC and to eliminating pyruvate from the network. The new chemical equation for vPDC is:

BPG_PEP+2ADP→ACA+2ATP.

**Figure 8:**
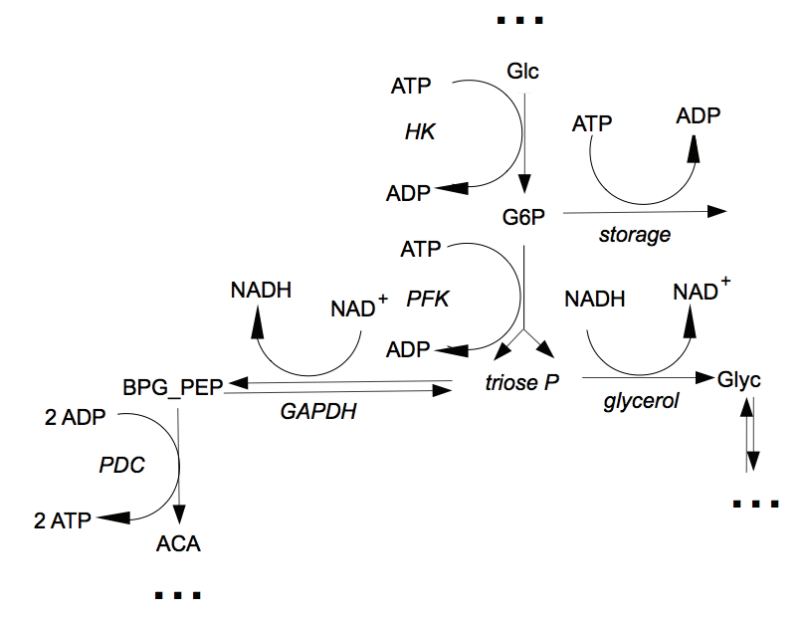
**Modified part of the reaction scheme for the reduced glycolysis model of *S. cerevisiae***.

In summary, after these four simplification steps the full model (original values in parentheses) has been reduced eventually to 17 (22) species and 19 (24) reactions with a total of 43 (59) parameters (the reduced reaction network is depicted on the Figure 8).

##### Third step: Parameter adjustment and verification of the reduced model

Due to the fact that the meaning of parameters has been changed in the course of model reduction these parameters (e.g. K4eq) need to be adjusted in order to obtain the full original behavior. This can be simply achieved by parameter scanning around the initial value. It is worth emphasizing here, that not all parameters have to be refitted, only the ones that result from the simplification of the lumping terms (e.g. quasi equilibrium constants resulting from the QEAs).

**Figure 9:**
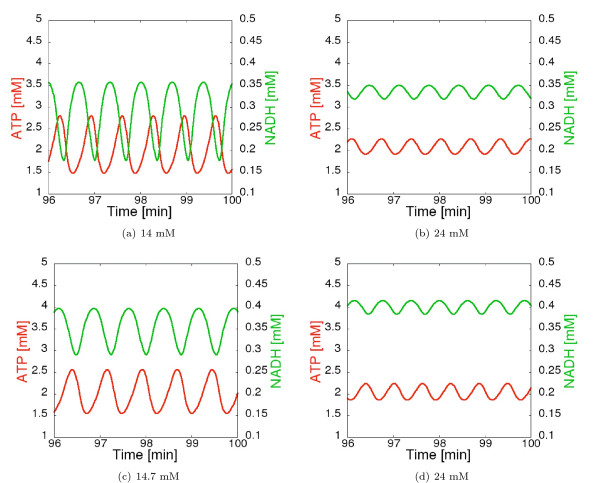
**Time courses of [ATP] and [NADH] in the two oscillating regimes at concentrations of [Glc*_x_*]_0 _as indicated**. The upper diagrams show the simulation of the full model, the lower the ones of the reduced system.

Finally we evaluate the reduced model by comparing its dynamic properties with the ones of the original full model. Comparative simulations are shown in Figure 9 and reveal that the reduced model captures the essential dynamics of the full model quantitatively very well - except for the amplitudes and the exact location of the bifurcation points for the first oscillatory regime. This discrepancy is of (only) quantitative nature and it does not occur if the full model is reduced by just three reactions (instead of five) as presented in Additional file 3.

## 3 Discussion and Conclusions

In this paper, we have presented a strategy for model simplification and reduction based on the CSP method. For this purpose and in order to make the method publicly available we implemented the original CSP algorithm in the COPASI software.

The CSP method is restricted to ODE models. Previously described simplification routines based on CSP mainly focus on the conversion of ODEs into DAE systems. In contrast, we use the CSP method to simplify models by lumping those reactions together that could be identified as being in QE. In addition, algebraic equations are used for species that are identified by Radical Pointers. Accordingly, we redefine chemical equations and kinetic rate laws of affected reactions. We demonstrated the usability of this approach using the COPASI implementation of the CSP method for a simple one-enzyme reaction and for a rather complex model of yeast glycolysis [16].

The time scale separation analysis of the glycolysis model revealed five reactions (vPGI, vALD, vTIM, vlpPEP, and vPK) for which the simplification strategy can be applied. We demonstrated that the resulting reduced model is capable of maintaining characteristics of the full model within an acceptable error range:

(i) same dynamic regimes, e.g. Hopf bifurcation point at [Glc*_x_*]_0 _= 18.5 mM; (ii) similar steady state levels of metabolite concentrations; (iii) similar periods for both and amplitudes for the second oscillatory regimes.

Studying different dynamics underlines again (as in [11]) the importance of time-resolved analyses since the contribution of the players in the system may vary over time and in between different dynamical regimes. This is ignored if either steady state data (or single time point data in general) or single dynamic regimes are studied.

Compared to our previous work on the ILDM method [10,11] - or the ILDM method in general - the CSP allows a more straightforward interpretation of its results with respect to the identification of QSS species and especially QE reactions. In addition, the Importance Index of CSP allows to analyze the impact of individual reactions on the dynamics of the species in the system.

An interesting outcome of our analysis is that it is possible to follow the general inherent temporal organization of the entire system when analyzing the distinctive time scales. Thus, we could observe that for the second oscillatory regime, all time scales oscillate in phase, partially overlapping each other which indicates that the whole system shows slower or faster dynamics in the course of a period.

Moreover, the number of fast modes changes over time and is also different for different dynamic regimes. Both factors prohibit the use of a fixed number of modes for time scale decomposition.

Furthermore, we suggest that the results of the CSP analysis can also be used for studying the relative importance of different reactions for the dynamics of the system. As an example, we observed that the overall participation of PFK in the slow modes increases with increasing glucose levels. In a simple way, this may be explained by the increasing energy charge (ATP concentration) which inhibits the PFK. Therefore, the relative importance of the PFK to the slower modes of the system increases.

Another beneficial result of the simplification process is of course that the number of system parameters is considerably reduced, especially concerning parameters which are involved in processes on a faster time scale than the time scale of interest which are then usually hard to identify. Therefore, using this process less system parameters will be unidentifiable.

Our study is not the first trying to reduce the original glycolysis model by [16]. [13] analyzed exclusively the limit cycle of the second oscillatory regime ([Glc*_x_*]_0 _= 24 mM) employing CSP without taking into account transient behavior. In contrast, we analyzed the model with original initial values taking into consideration also the initial transient time period. In addition, there are major methodological differences. First, our approach focuses on simplifying the underlying biochemical reaction network rather than on approximating the ODE system with a DAE system. Second, we do not fix the number of fast modes. Third, we compute the normed sum of PIs over the entire fast subspace in order to justify QEA.

A completely different approach was taken by [22]. Their sole criterium for the reduction was the fulfillment of a Stuart-Landau equation which is in principle only valid in the vicinity of a Hopf bifurcation and therefore does not offer a general strategy for system reduction.

Obviously, there are some relations between CSP output data and sensitivity analyses like metabolic control analysis (MCA). Learning e.g. about the impact of individual reactions on systems properties like dynamics could in principle also result from sensitivity analyses. We did a preliminary comparison of the results of our CSP analysis and a conventional MCA for the steady state. This resulted in a similar global picture, but the CSP gave a more fine-grained picture w.r.t. the relative importance of reactions on species. In addition, the time-resolved analysis for oscillations is not possible with MCA.

With all the mentioned benefits of using CSP for systems analysis, there are also problems and limitations arising from this approach. We employed several heuristic thresholds for the discrimination of the reactions and species mainly contributing to the fast subspace of the system. These were based on our experience and obviously, this might not be optimal for arbitrary systems. Thus, other systems might demand slightly altered thresholds. This is underlined by the fact that we observed one reaction - AK - that in principle fulfilled all of our criteria for elimination, but in the end, it turned out to be impossible to eliminate from the system without introducing a large error. Therefore, it is always important to carefully check the behavior of the reduced system. The CSP can only support this process in a rational way, but does not allow for a fully automated analysis.

Even though, accordingly, scientists will always have to be on top of this method, it would be useful to support the reduction of the system in a stronger way than just providing the CSP. A semi-automated reduction which then quickly allows to be checked for error compared to the original model would reduce workload considerably and is currently planned to be included in the software. An additional planned extension of the software is the support of different compartment sizes (if multi-compartment models are analyzed) which is currently not the case.

All in all, we were surprised that taking into account different dynamic regimes only allowed the elimination of 5 reactions and 5 species of the glycolysis model which is considerably less than previous attempts that focused on particular regimes. This once again supports the view that it is crucial to define which systems behaviors should be reproduced by the simplified model before entering reduction strategies and these initial decisions might result in different models in the end.

## Competing interests

The authors declare that they have no competing interests.

## Authors' contributions

IS conceived the procedure for CSP based model reduction, performed the CSP analysis and drafted the initial manuscript. NS adapted the CSP for COPASI and implemented the algorithm. KH analyzed the CSP data, classified and interpreted it biochemically. SS supported the method implementation. UK initiated the project and interpreted the results biochemically. All authors participated in discussions and writing of the final manuscript. All authors also read and approved the final manuscript.

## Supplementary Material

Additional file 1 **Michaelis Menten Kinetics**: Includes a more detailed simplification procedure of Michaelis Menten kinetics. Click here for file

Additional file 2 **CSP output data for glycolysis model**: Includes the complete set of CSP output data (time resolved TS, RP, PI and II) for the glycolysis model. Click here for file

Additional file 3 **simplification of glycolysis model**. Additional material related to the simplification of the glycolysis model. This includes a list of original and modified reactions, kinetics laws and parameters. Click here for file
