# Supplementary material for: Simplification of biochemical models: a general approach based on the analysis of the impact of individual species and reactions on the systems dynamics
Source: BMC Syst Biol. 2012 Mar 5;6:14. doi: 10.1186/1752-0509-6-14 (PMC3349553; doi:10.1186/1752-0509-6-14)
Supplement: Additional file 1 — Michaelis Menten Kinetics: Includes a more detailed simplification procedure of Michaelis Menten kinetics. [file 1752-0509-6-14-S1.PDF]

Electronic Supplementary Information

for

Simplification of biochemical models based on the analysis of the impact of individual species and reaction on the systems dynamics: a general approach.

Irina Surovtsova, Natalia Simus, Katrin Hübner, Sven Sahle and Ursula Kummer

Michaelis Menten Model

In order to illustrate the handling of the CSP based model reduction we consider here in more details the simplest enzymatic reaction mechanism, the irreversible Michaelis–Menten kinetics:

$$\underset{\text{Substrate}}{S} + \underset{\text{Enzyme}}{E} \underset{k_{-1}}{\overset{k_1}{\Longleftrightarrow}} \underset{\text{Complex}}{C} \xrightarrow{k_2} \underset{\text{Product}}{P} + \underset{\text{Enzyme}}{E}.$$

This leads to following differential Equations:

$$\begin{aligned}
\frac{dS}{dt} &= -k_1 \cdot S \cdot E + k_{-1} \cdot C \\
\frac{dC}{dt} &= k_1 \cdot S \cdot E - (k_2 + k_{-1}) \cdot C \\
\frac{dP}{dt} &= k_2 \cdot C \\
\frac{dE}{dt} &= -k_1 \cdot S \cdot E + (k_{-1} + k_2) \cdot C
\end{aligned}$$

which can be further simplified taking into account enzyme conservation: $E_0 = E(t) + C(t)$ (with initial enzyme concentration E_0)

$$\begin{aligned}
\frac{dS}{dt} &= -k_1 \cdot E_0 \cdot S + (k_1 \cdot S + k_{-1}) \cdot C \\
\frac{dC}{dt} &= k_1 \cdot E_0 \cdot S - (k_1 \cdot S + k_2 + k_{-1}) \cdot C \\
\frac{dP}{dt} &= k_2 \cdot C
\end{aligned}$$

As in article we consider two limit situations for the dimensionless parameters:

$$S_t = \frac{k_2}{k_{-1}} \to 0 \text{ and } M_r = \frac{E_0}{S_0} \to 0$$

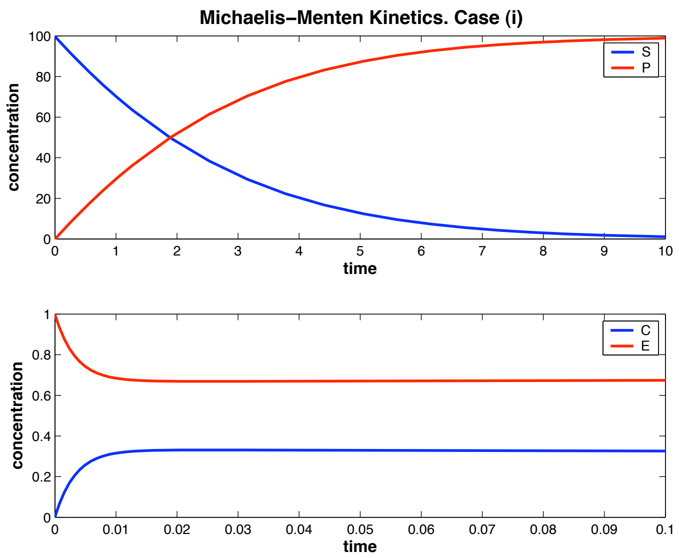

Figure 1: Simulation of Michaelis Menten model via Matlab. $M_r \to 0$. Calculations for $S_0 = 100, E_0 = 1, k_1 = 1, k_{?1} = k_2 = 100$)

(here S_0 is initial substrate concentrations).

(i) $\mathbf{M_r \to 0}$ $(\mathbf{E_0 \ll S_0})$ (Fig 1 displays the model simulation via Matlab)

As described in the main article the CSP method allows the distinction between slow and fast modes (for times $t > 0.03$). The Radical Pointer from the CSP data shows that the complex C dominates the fast mode. The contributions of both reactions to the slow and fast modes are comparable (see figure 1(a) in the main article, which displays the evolution in time of Radical Pointer and Participation Indices). Thus, the QSSA for the complex C is justified in this case and leads to the Michaelis-Menten kinetic law:

$$\frac{dC}{dt} \approx 0, \qquad \frac{dS}{dt} = -k_2 \cdot \frac{E_0 \cdot S}{S + K_m}$$

$$C = \frac{E_0 \cdot S}{S + K_m}, \text{ where } K_m = \frac{k_{-1} + k_2}{k_1}$$

(ii) $\mathbf{S_t \to 0}$ $(\mathbf{k_2 \ll k_{-1}}, \quad \mathbf{E_0 = S_0})$. (s. Fig 2 for model simulation.)

This limit means that an equilibrium between the enzyme E, the substrate S and the enzyme–substrate complex C is established quickly. The slow step is the breakdown of C to produce the product P and the enzyme E.

The CSP analysis leads to the occurrence of two independent dynamical modes. After the short transient phase ($t < 0.006$, when no reduction is possible) the contribution of C to the fast mode is larger than the one of S (nevertheless no real dominance occurs). During the time the contribution of both variables becomes equal (s. Fig. 1(b) in article). Thus, the QSSA for complex C is incorrect.

Nevertheless, there is a clear separation of reactions in the modes. The reaction of product formation dominates clearly the slow mode. Both reactions are active in the fast mode (s. Fig.1(b) in the main text). Thus, the reaction: $S + E \underset{k_{-1}}{\overset{k_1}{\Longleftrightarrow}} C$ is always practically in equilibrium and the

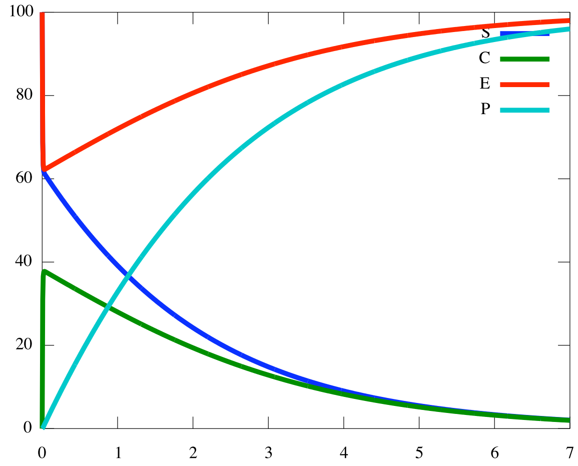

Figure 2: Simulation of Michaelis Menten model via Matlab. $S_t \to 0$. Calculations for $S_0 = 100, E_0 = 100, k_1 = k_2 = 1, k_{?1} = 100$)

QEA for this is correct and leads to a similar equations as for "standard" Michaelis-Menten kinetics:

$$k_1 \cdot (E_0 - C) \cdot S \approx k_{-1} \cdot C$$

$$C = \frac{E_0 \cdot S}{S + K_s}, \qquad \text{with equilibrium constant } K_s = \frac{k_{-1}}{k_1}$$

$$\frac{d\,P}{d\,t} = \frac{k_2\,E_0\,S}{K_s + S}.$$
