# Supplementary material for: Simplification of biochemical models: a general approach based on the analysis of the impact of individual species and reactions on the systems dynamics
Source: BMC Syst Biol. 2012 Mar 5;6:14. doi: 10.1186/1752-0509-6-14 (PMC3349553; doi:10.1186/1752-0509-6-14)
Supplement: Additional file 2 — CSP output data for glycolysis model: Includes the complete set of CSP output data (time resolved TS, RP, PI and II) for the glycolysis model. [file 1752-0509-6-14-S2.PDF]

Electronic Supplementary Information
for

Simplification of biochemical models based on the analysis of the impact of individual species and reaction on the systems dynamics: a general approach.

Irina Surovtsova, Natalia Simus, Katrin Hübner, Sven Sahle and Ursula Kummer

Glycolysis in Saccharomyces cerevisiae (Hynne 2001)

Here we present the CSP output data as described in the article (Time Scales, Radical Pointer, Participation Index and Importance Index). The CSP analysis was performed for three different dynamical regimes ($\text{GlcX}_0 = 24, 14$ and 9 mM). For each CSP analysis we simulate the system for a time period from 0 to 100 min and inspect 250 time points along the trajectory. At each time point a full set of CSP data is computed. The CSP parameters *Ratio of mode separation*, *Relative Error* and *Absolute Error* are set to 0.9, 1e-3 and 1e-4, respectively.

In the following pictures the right, middle and left columns correspond to the steady state regime ($\text{GlcX}_0 = 9mM$), the first ($\text{GlcX}_0 = 14mM$), and second oscillatory regimes ($\text{GlcX}_0 = 24mM$) respectively.

1 Time scales: Hynne 24 - Hynne 14 - Hynne 9

- The time scales of the first 6 fast modes are well separated from the slow modes:

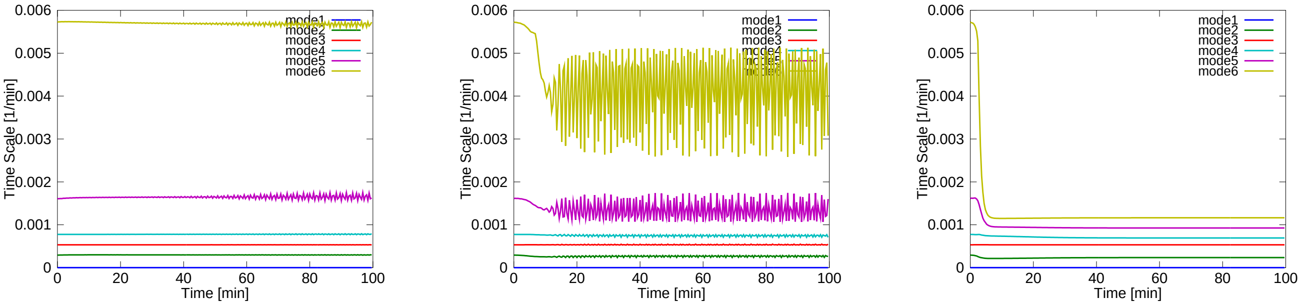

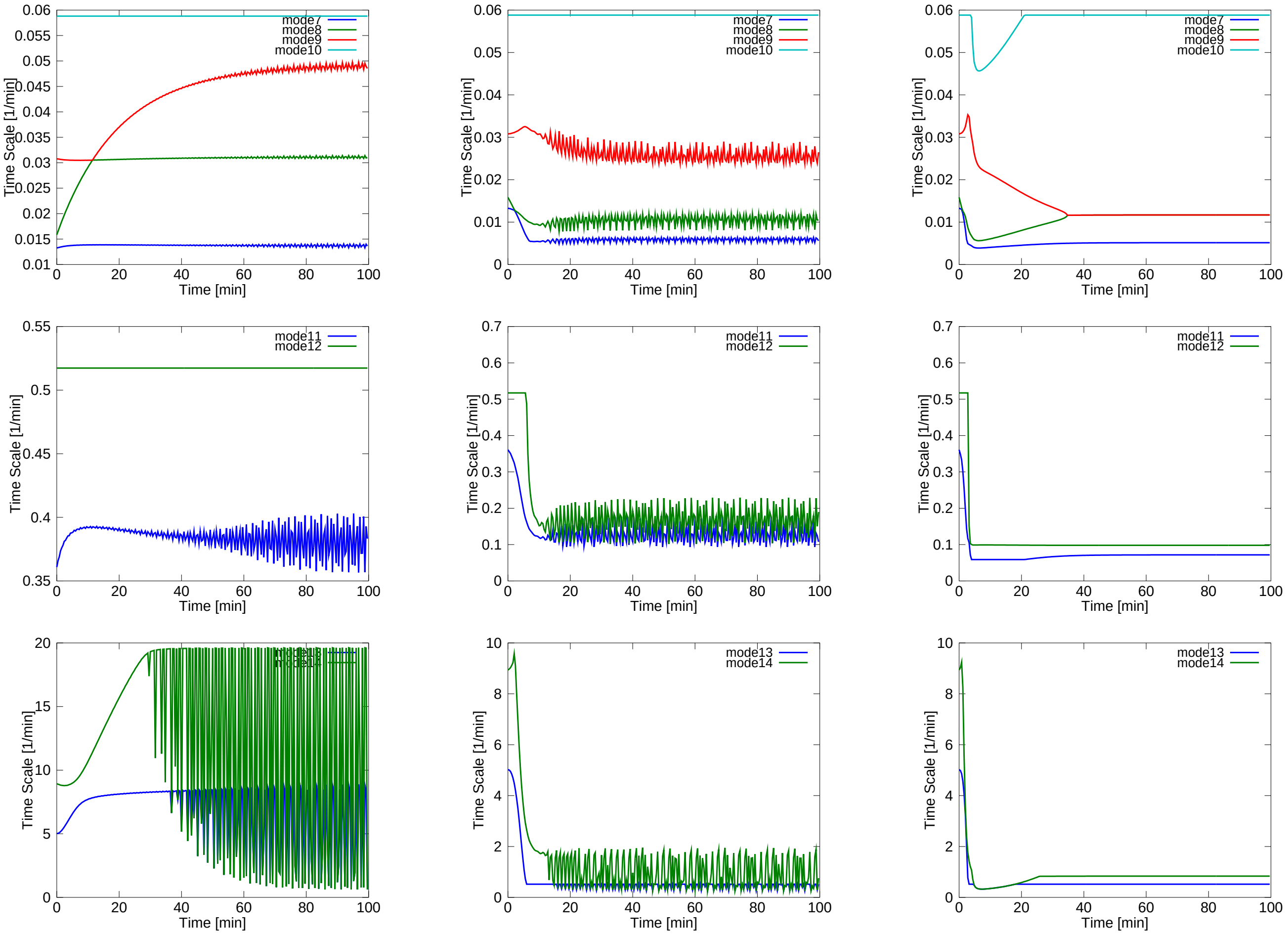

- The time scales of mode 15-18 show wide variation for the oscillating regime (Hynne-24).

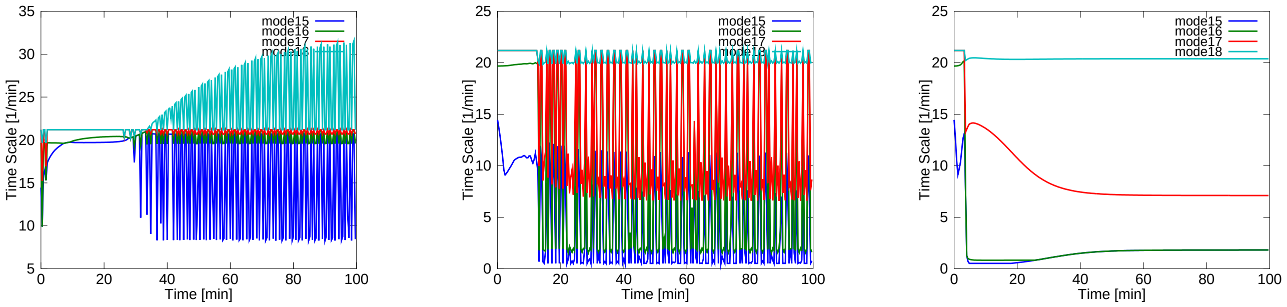

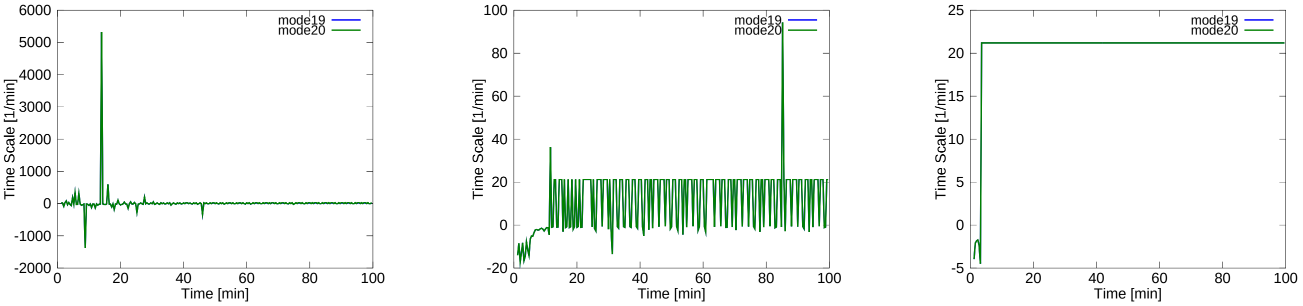

2 Radical Pointer: Hynne 24 - Hynne 14 - Hynne 9

- The Radical Pointer is used to indentify QSS metabolites (the species with large radical pointer could be described as being in a QSS).
- More specifically, we define the metabolite as being in a QSS if the sum of CSP radicals over all fast modes is larger than 0.9.
- The graphs below display these sums for the different metabolites.

The right, middle and left columns correspond to the steady state regime ($\mathrm{GlcX}_0 = 9mM$), the first ($\mathrm{GlcX}_0 = 14mM$), and second oscillatory regimes ($\mathrm{GlcX}_0 = 24mM$) respectively.

Metabolites ACA, Glc, Glyc and EtOH

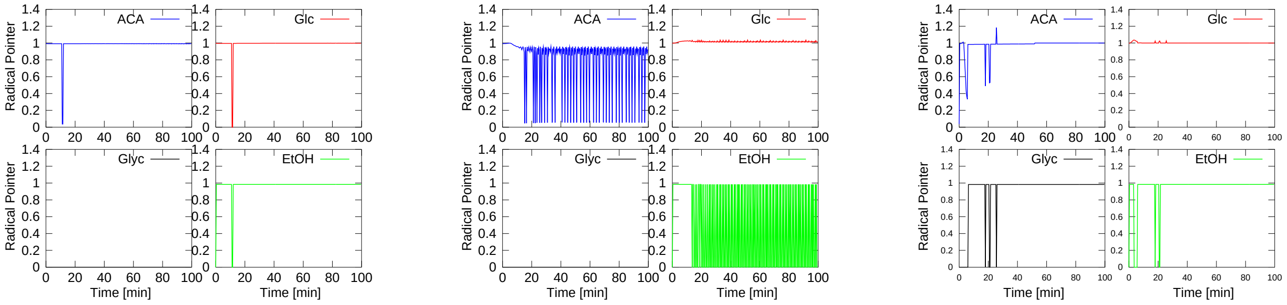

Metabolites ADP, GAP, DHAP and G6P

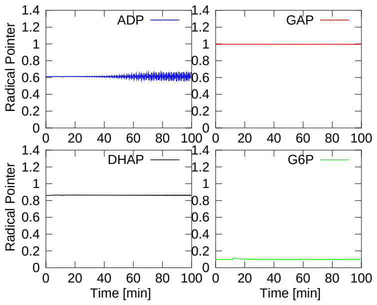

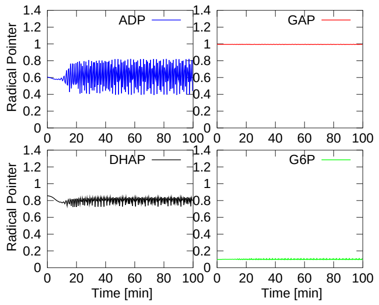

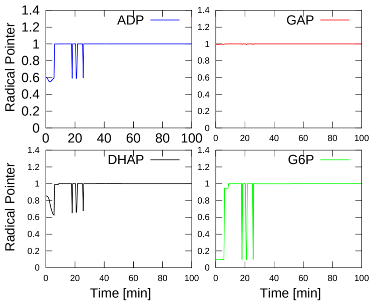

Metabolites CNX, Pyr, EtOHX and GlycX

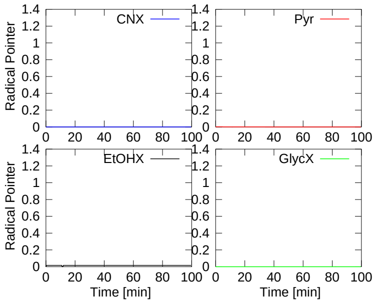

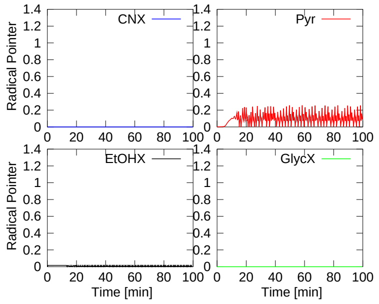

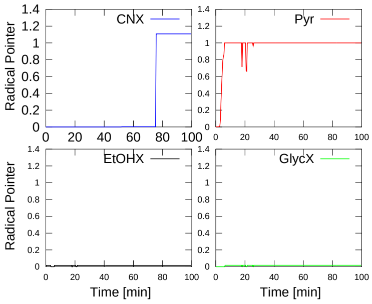

Metabolites GlcX, BPG, FBP and AMP

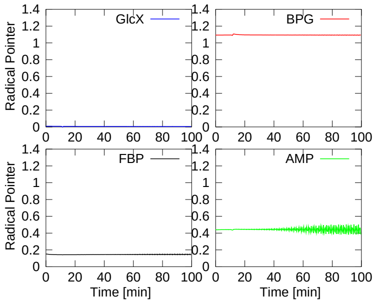

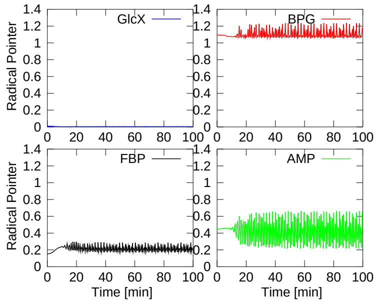

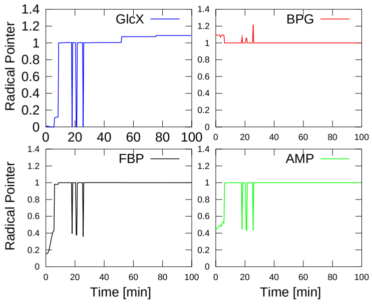

Metabolites NAD, ACAX, PEP and F6P

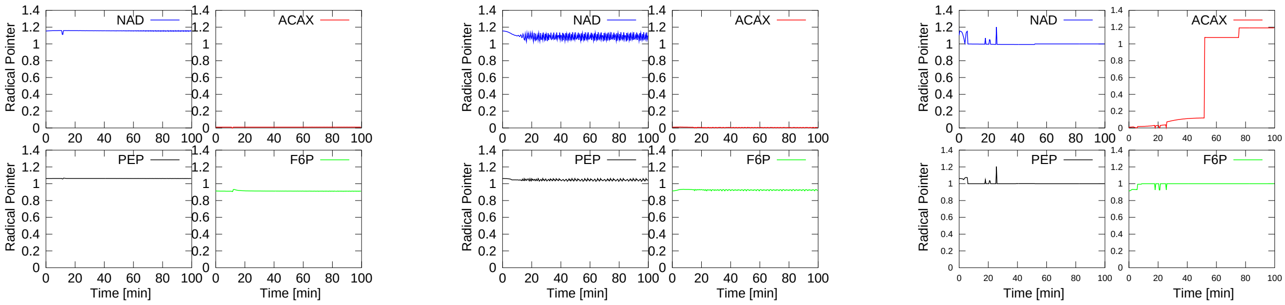

3 Participation Index (PI): Hynne 24 - Hynne 14 - Hynne 9

We now examine the participation indices as a function of time for the three dynamical regimes of the model. On the following graphs we show the normed sums of the Participation Indices over the fast and slow subspaces, respectively. We define a reaction as fast if its normed contribution over all fast modes is larger as 0.7

The right, middle and left columns correspond to the steady state regime ($\text{GlcX}_0 = 9mM$), the first ($\text{GlcX}_0 = 14mM$), and second oscillatory regimes ($\text{GlcX}_0 = 24mM$) respectively.

Reactions vinGlc (left top), vHK (right top), vPGI (left bottom), vPFk (right bottom)

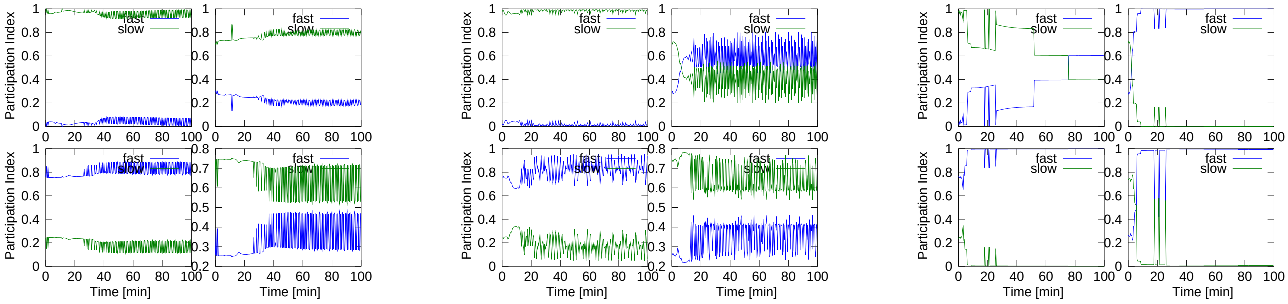

- Investigation of the PI for first four reactions reveals that the glucose input reaction vinGlc is a slow reaction, whereas the PGI reaction is fast for all dynamic regimes. We note that the HK and PFK reactions are slow for the oscillating regimes, but not for the case $[\text{Glc}_x]_0 = 9$, where the reactions are slow at the beginning and fast for time $t > 10$.

Reactions vALD (left top), vTIM (right top), vGAPDH (left bottom), vlpPEP (right bottom)

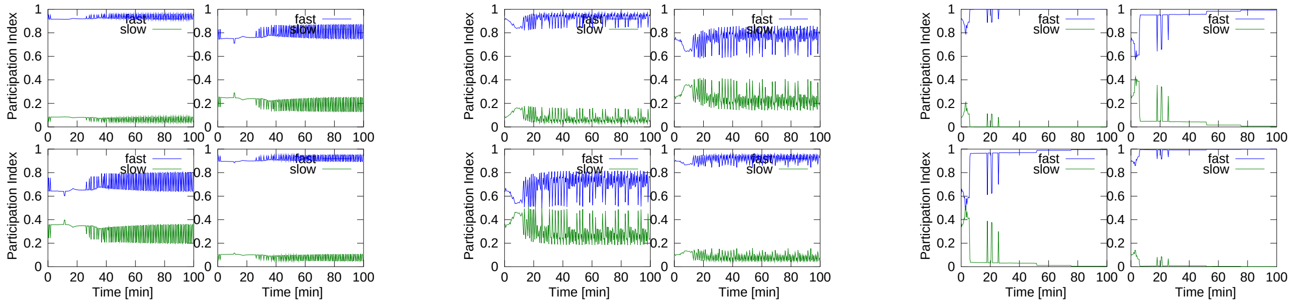

The ALD and PeP reactions are clearly fast reactions. More complicated are GAPDH and TIM reactions, which are both mainly fast, but also participate in the slow space.

Reactions vPK (left top), vPDC (right top), vADH (left bottom), vinCN (right bottom)

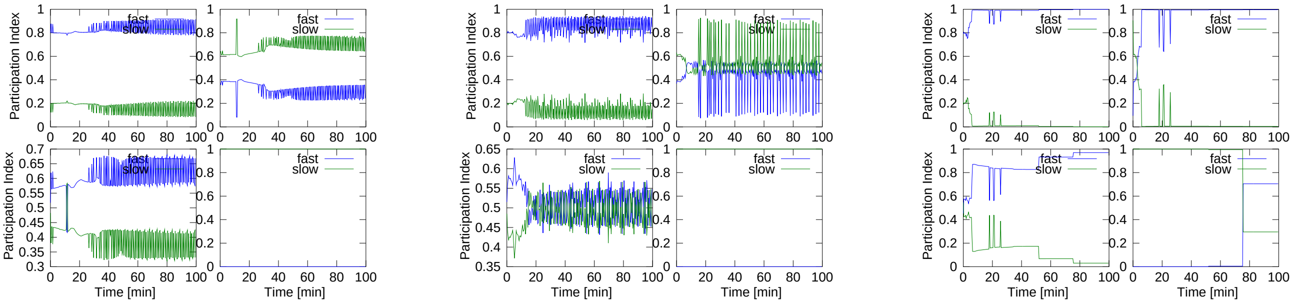

The PK is a fast reaction. Reaction inCN is slow. The time evolution of the ADH reaction's participation index shows complicated behavior (the reaction is active in both fast and slow space). PDC is participates more in the slow subspace during oscillation and more in the fast subspace for the steady state.

Reactions voutEtOH (left top), vlpGlyc (right top), voutGlyc (left bottom), voutACA (right bottom)

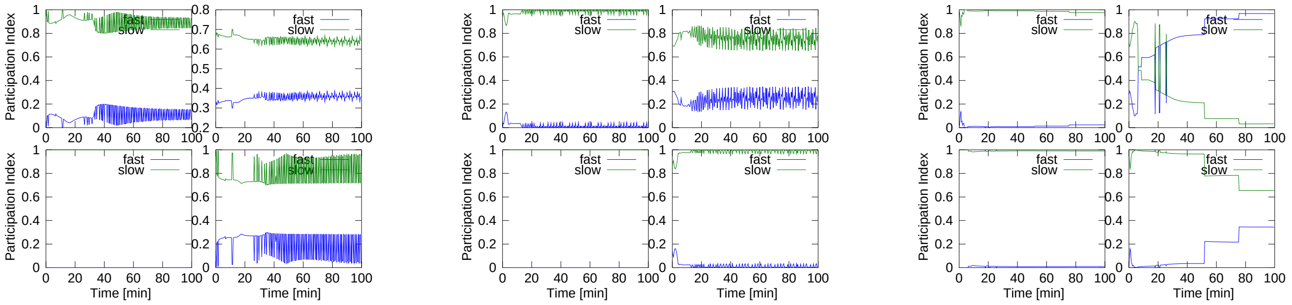

All four reactions here are clearly slow.

Reaction vstorage (left top), vconsum (right top), vGlcTransI (left bottom), vlacto (right bottom)

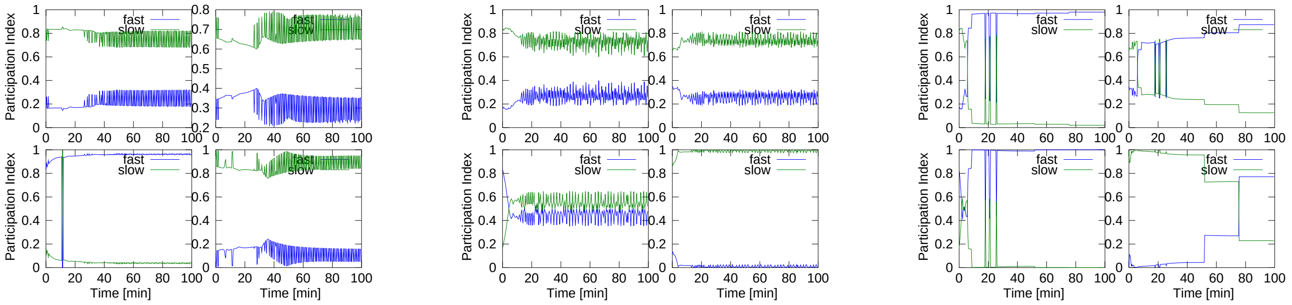

The glucose transport (GlcTransI) is a fast reaction for the second oscillatory regime ($[\mathrm{Glc}_x]_0 = 24$) and in the case $[\mathrm{Glc}_x]_0 = 9$. The other three reactions are slow.

Reaction vAK (left top), vdifACA (right top), vGdifGlyc (left bottom), vdifEtOH (right bottom)

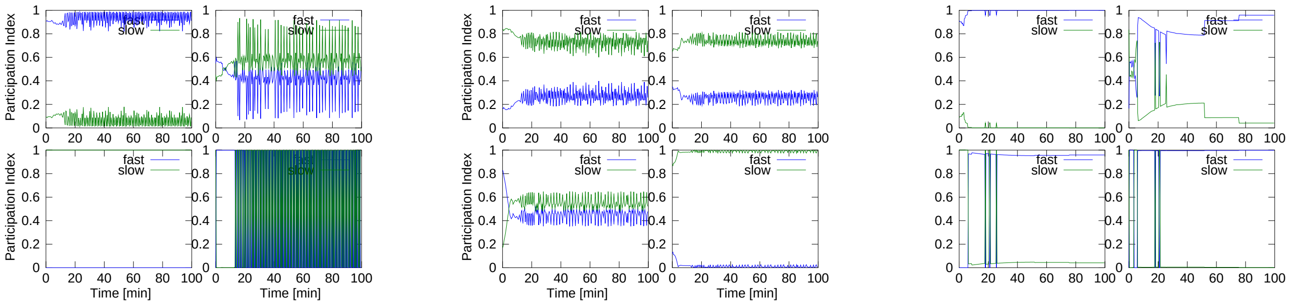

AK is fast reaction.

Conclucion from PI analysis:

- There are 5 clearly fast reactions in the system: PGI, ALD, PEP, PK and AK
- Two other reactions (TIM and GAPDH) could also be approximated as being in QE. Nevertheless the reduction should be done more carefully.
- Most interesting are the HK and glucose transporter (GlcTransI) reactions, for which the PI time evolution shows clearly different behavior for oscillating and steady state regimes.

3.1 Importance Index (II): Hynne 24 - Hynne 14 - Hynne 9

The Importance Index (II) describes the relative importance of the contribution of the r-th reaction to the rate of change of the i-th element of concentration vector **y**. The following pictures represent Importance Indexes of all 24 reactions of the initial model. The distinct graphs correspond to the distinct elements of concentration vectors according the following color legend:

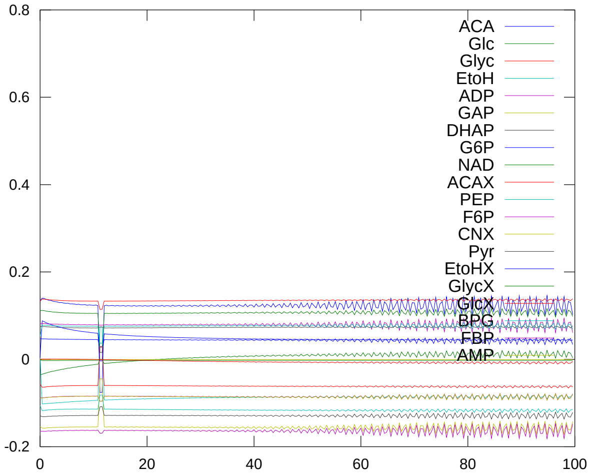

- We indentified 9 reactions as having the largest Importance Indices: HK, PFK, GAPDH, PDC, ADH, outEtoH, lpGlyc, outGlyc and storage. These reactions exhibit the most significant influence on the system dynamics.

Reactions vinGlc (left top), vHK (right top), vPGI (left bottom), vPFk (right bottom)

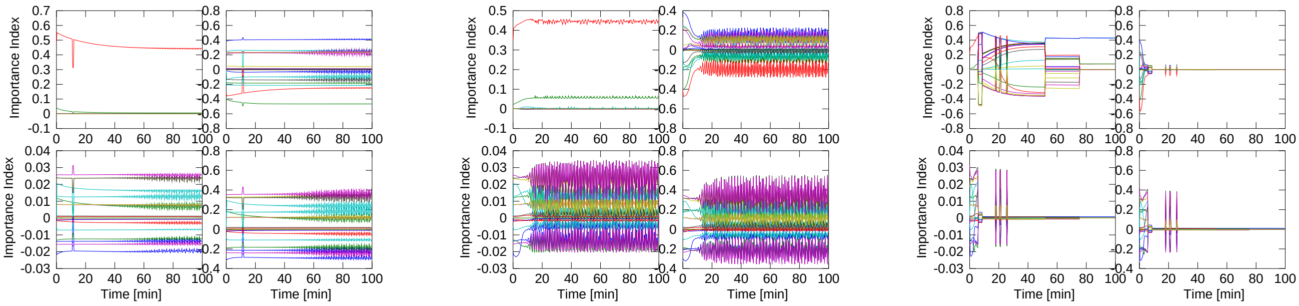

- The reaction catalyzed by PFK is important for controlling the system dynamics.
- The reaction catalyzed by HK controls most metabolites during oscillations, but plays no significant role for the system in the steady state regime.
- In the oscillating case the Importance Index of membrane transport reaction inGlc is only significant for GlcX. In the steady state the Importance Indices are significant for almost all species for time $7.1 < t < 10.9$:

Reactions vALD (left top), vTIM (right top), vGAPDH (left bottom), vlpPEP (right bottom)

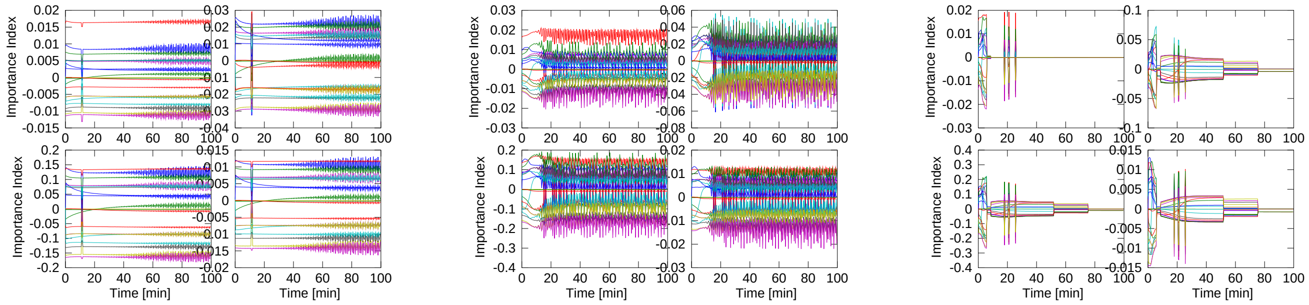

- The GAPDH reaction has large II values.

Reactions vPK (left top), vPDC (right top), vADH (left bottom), vinCN (right bottom)

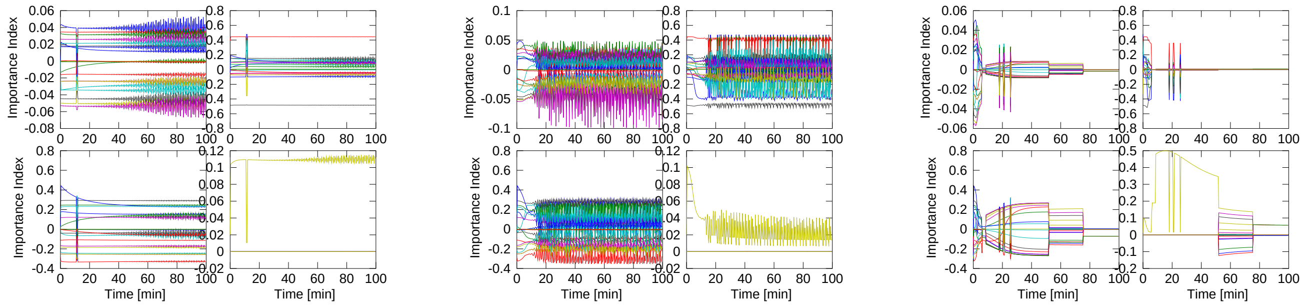

- The IIs are large for the PDC and ADH reactions.
- According to IIs, the PDC reaction has large impact on ACAX and PYR during oscillations (positive value about 0.4) and PYR (with negative value of about -0.5).
- For inCN reaction: CNX has most significant II.

Reactions voutEtOH (left top), vlpGlyc (right top), voutGlyc (left bottom), voutACA (right bottom)

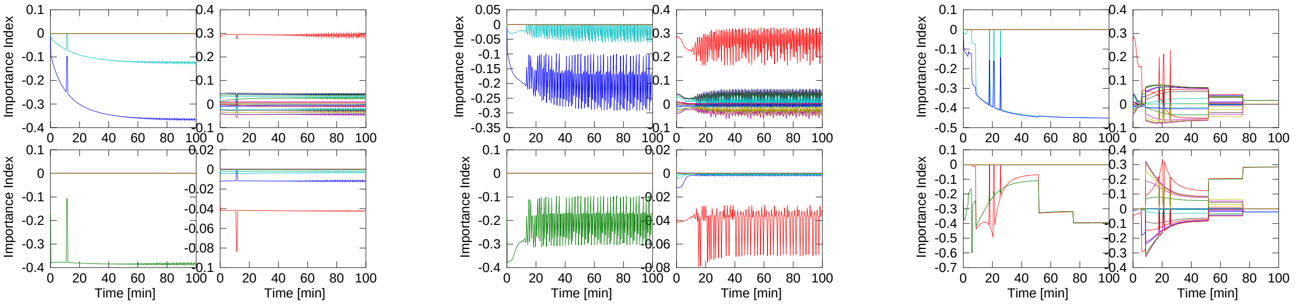

- outEtOH reaction: most significant IIs are for EtoH (about -0.1) and EtoHX (with negative value of about -0.4).
- For lpGlyc reaction: Glyc has most significant II.
- For outGlyc reaction: GlycX has most significant II.
- For outACA reaction: most significant IIs are for ACA (about -0.01) and ACAX (with negative value of about -0.04).

Reaction vstorage (left top), vconsum (right top), vGlcTransI (left bottom), vlacto (right bottom)

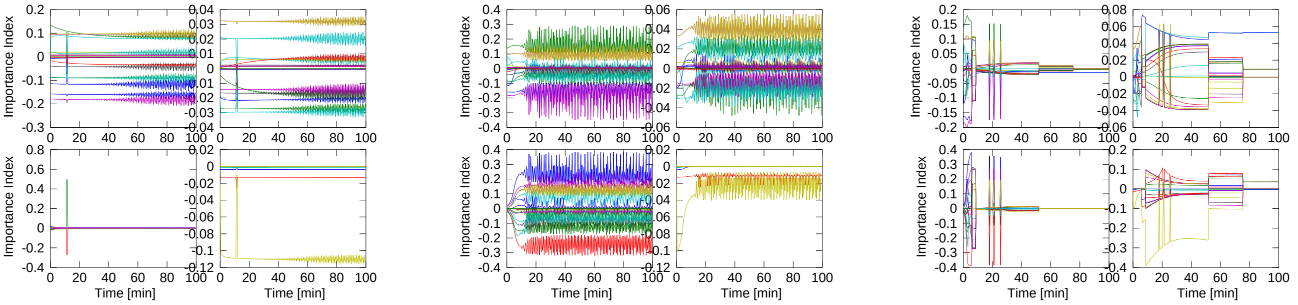

- For lacto reaction: most significant IIsare for CNX (about -0.12, yellow line) and ACAX (with negative value of about -0.02, red line).
- The glucose transporter GlcTransI plays no significant role for any species during oscillations. For the steady state regime this reaction controls most of metabolites.

Reaction vAK (left top), vdifACA (right top), vGdifGlyc (left bottom), vdifEtOH (right bottom)

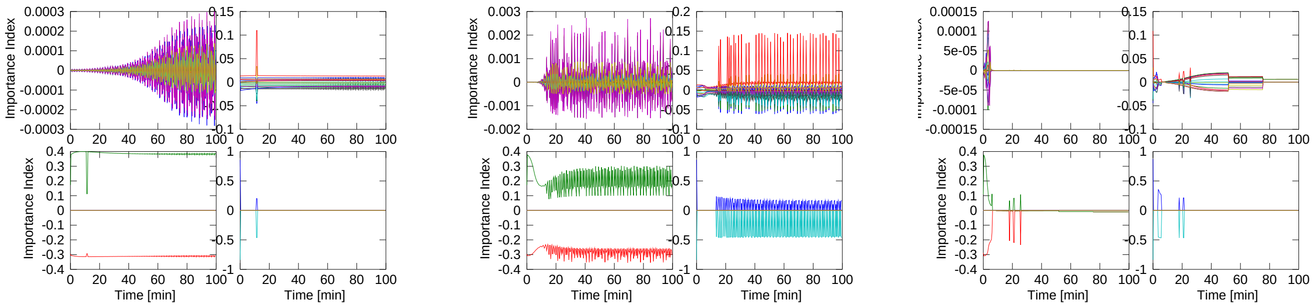

- The most significant Importance Indices in difGlyc reaction have GlycX (about 0.4, green line) and Glyc (with negative value of about -0.3, red line)
