# Supplementary material for: Simplification of biochemical models: a general approach based on the analysis of the impact of individual species and reactions on the systems dynamics
Source: BMC Syst Biol. 2012 Mar 5;6:14. doi: 10.1186/1752-0509-6-14 (PMC3349553; doi:10.1186/1752-0509-6-14)
Supplement: Additional file 3 — simplification of glycolysis model. Additional material related to the simplification of the glycolysis model. This includes a list of original and modified reactions, kinetics laws and parameters. [file 1752-0509-6-14-S3.PDF]

Electronic Supplementary Information
for
Simplification of biochemical models based on the analysis of the impact of individual species and reaction on the systems dynamics: a general approach.

Irina Surovtsova, Natalia Simus, Katrin Hübner, Sven Sahle and Ursula Kummer

Glycolysis in Saccharomyces cerevisiae (Hynne 2001)

Model Reduction

3–reaction reduced model: Elimination of PGI, ALD and TIM

vPGI: G6P = F6P **is fast reaction**

The QEA leads to following algebraic equation:

$$K_{\mathrm{PGI}} \approx \frac{\mathrm{F6P}}{\mathrm{G6P}}.$$

here K_{PGI} is equilibrium constant. Its original value is given in table 1.

In order to eliminate F6P from the system and to lump PGI together with PFK we need to modify the chemical equation of the PFK reaction to

$$\mathrm{G6P} + \mathrm{ATP} \rightarrow \mathrm{FBP} + \mathrm{ADP}$$

as well as the kinetic rate law of PFK to

$$\frac{V_{5m} \cdot (K_{PGI} \cdot G6P)^2}{\left(K_5 \cdot \left(1 + \kappa_5 \cdot \left(\frac{ATP}{AMP}\right)\right)^2 + (K_{PGI} \cdot G6P)^2\right)}.$$

vALD: FBP = GAP + DHAP **and vTIM:** GAP = DHAP **are fast reactions**

The QEAs lead to two algebraic equations:

$$\mathrm{FBP} \approx \frac{\mathrm{GAP} \cdot \mathrm{DHAP}}{K_{6eq}}, \qquad V_{7f} \cdot \mathrm{GAP} \approx V_{7r} \cdot \frac{\mathrm{DHAP}}{K_{7eq}}$$

We introduce the pool metabolite trioseP:

Kinetic Parameter (Reaction)	κ (PFK)	$K_{4eq}(PGI)$	K_{6eq} (ALD)	K_{7eq} (TIM)	$GAP_{st}(ALD)$
Original value	0.15	0.13	0.081	0.055	0.12
Values in reduced model	0.124	0.11	0.02296	0.055	0.1157

Table 1: Modified kinetic parameters in 3–reaction reduced model

$$\text{trioseP} = \text{GAP} + \text{DHAP} + \text{FBP} = \text{GAP} \cdot \left[1 + \frac{1}{K_{7eq}} + \frac{\text{GAP}_{st}}{K_{7eq} \cdot K_{6eq}}\right] \equiv \text{GAP} \cdot K$$

In order to solve the algebraic equation for trioseP we simplified the value in the bracket fixing GAP at its steady state value. Then we reduce vALD and vTIM from the reaction network.

$$\begin{aligned} \text{GAP} &= \frac{\text{trioseP}}{K} \\ \text{DHAP} &= \frac{\text{trioseP}}{K_{7eq} \cdot K} \\ \text{FBP} &= \frac{\text{trioseP}^2}{K_{7eq} \cdot K_{6eq} \cdot K} \end{aligned}$$

The corresponding branch of the reaction network:

$$\begin{aligned} R5(PFK): &\quad \text{F6P} + \text{ATP} \rightarrow 2 \cdot \text{trioseP} + \text{ADP} \\ R8(GAPDH): &\quad \text{trioseP} + \text{NAD} = \text{BPG} + \text{NADH} \\ R15(Glyc): &\quad \text{trioseP} + \text{NADH} = \text{Glyc} + \text{NAD} \end{aligned}$$

Figures 1 and 2 show the simulation of the full model and reduced model (by eliminating three QE reactions: PGI, ALD and TIM) for both oscillatory regimes $\text{GlcX}_0 = 14$ and $\text{GlcX}_0 = 24$ respectively. Comparative simulations reveal that the 3–reaction reduced model captures the dynamics of the original model (with light increasing of oscillation period only) very well. The refitted kinetic parameters of modified reactions are displayed in table 1.

5–reaction reduced model: Elimination of PGI, ALD, TIM, vlPEP and PK

vlPEP: BPG + ADP = PEP + ATP **and PK:** PEP + ADP = PYR + ATP **are fast reactions**

The QEA for the lpPEP reaction leads to following algebraic equation:

$$k_{9f} \cdot \text{BPG} \cdot \text{ADP} - k_{9r} \cdot \text{PEP} \cdot \text{ATP} \approx 0$$

We introduce the "pool metabolite:"

$$\text{BPG\_PEP} = \text{BPG} + \text{PEP}$$

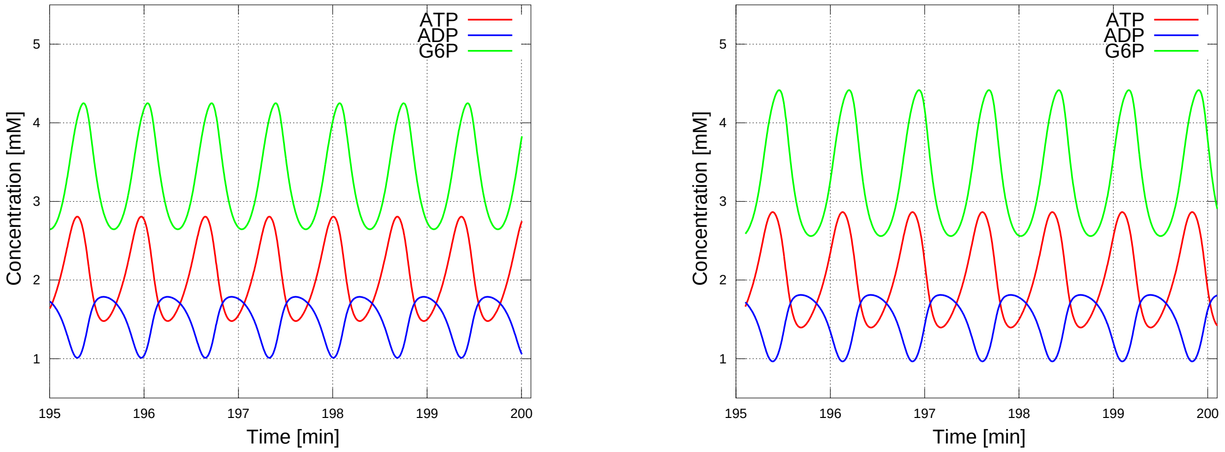

Figure 1: Time course of [ATP], [ADP] and [G6P] for $[\mathrm{GlcX}_0] = 14$. The left graph shows the time simulation of the full model, the right one of the reduced system

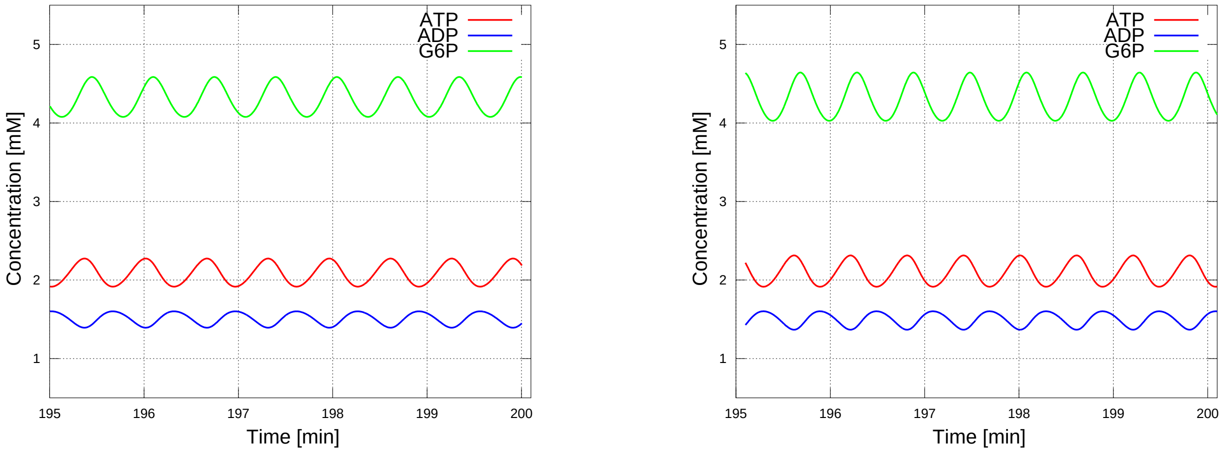

Figure 2: Time course of [ATP], [ADP] and [G6P] for $[\mathrm{GlcX}_0] = 24$. The left graph shows the time simulation of the full model, the right one of the reduced system

Reaction Name	Reaction in the Original Model	Reaction in the Reduced Model
PGI	G6P = F6P	
PFK	G6P + ATP → FBP + ADP	G6P + ATP → 2 · trioseP + ADP
ALD	FBP = GAP + DHAP	
TIM	GAP = DHAP	
GAPDH	GAP + NAD = BPG + NADH	trioseP + NAD = BPG_PEP + NADH
lpPEP	BPG + ADP = PEP + ATP	
PK	PEP + ADP = Pyr + ATP	
PDC	Pyr = ACA	BPG_PEP + 2 · ADP = ACA + 2 · ATP
Glyc	DHAP + NADH = Glyc + NAD	trioseP + NADH = Glyc + NAD

Table 2: Changed reactions in original and 5–reaction reduced model of glycolysis.

and reduce the vlPEP reaction from the network.

$$\mathrm{BPG} = \frac{\mathrm{BPG\_PEP} \cdot \mathrm{k_{9r}} \cdot \mathrm{ATP}}{\mathrm{k_{9f}} \cdot \mathrm{ADP} + \mathrm{k_{9r}} \cdot \mathrm{ATP}}$$
$$\mathrm{PEP} = \frac{\mathrm{BPG\_PEP} \cdot \mathrm{k_{9f}} \cdot \mathrm{ADP}}{\mathrm{k_{9f}} \cdot \mathrm{ADP} + \mathrm{k_{9r}} \cdot \mathrm{ATP}}$$

The new chemical equations of the associated reactions are given as:

$$R8(GAPDH): \quad \mathrm{GAP} + \mathrm{NAD} = \mathrm{BPG\_PEP} + \mathrm{NADH}$$
$$R11(PDC): \quad \mathrm{BPG\_PEP} + 2 \cdot \mathrm{ADP} = \mathrm{ACA} + 2 \cdot \mathrm{ATP}$$

The comparison between reactions in original and reduced model is given in table 2.
Table 3 refers to the initial kinetic laws which are changed by model simplification.
The modified kinetic laws are given in table 4.
The values of modified kinetic parameters are displayed in table 5.

The comparison of the full and 5–reaction reduced model is made in article.

Reaction Name	Kinetic Law in the Original Model
PGI	$\dfrac{V_{4f} \cdot \mathrm{G6P} - V_{4r} \cdot \dfrac{\mathrm{F6P}}{K_{4eq}}}{K_{4G6P} + \mathrm{G6P} + \dfrac{K_{4G6P}}{K_{4F6P}} \cdot \mathrm{F6P}}$
PFK	$\dfrac{V_{5m} \cdot \mathrm{F6P}^2}{\kappa_5 \cdot \left(1 + \kappa_5 \cdot \left(\dfrac{\mathrm{ATP}}{\mathrm{AMP}}\right)^2\right) + \mathrm{F6P}^2}$
ALD	$\dfrac{V_{6f} \cdot \mathrm{FBP} - \dfrac{V_{6f} \cdot \mathrm{GAP} \cdot \mathrm{DHAP}}{K_{6eq}}}{K_{6FBP} + \mathrm{FBP} + \dfrac{\mathrm{GAP} \cdot K_{6DHAP}}{K_{6eq} \cdot r_6} + \dfrac{\mathrm{DHAP} \cdot K_{6GAP}}{K_{6eq} \cdot r_6} + \dfrac{\mathrm{FBP} \cdot \mathrm{GAP}}{K_{6IGAP}} + \dfrac{\mathrm{GAP} \cdot \mathrm{DHAP}}{K_{6eq} \cdot r_6}}$
TIM	$\dfrac{V_{7f} \cdot \mathrm{DHAP} - \dfrac{V_{7r}\mathrm{GAP}}{K_{7eq}}}{K_{7DHAP} + \mathrm{DHAP} + \dfrac{K_{7DHAP}}{K_{7GAP}} \cdot \mathrm{GAP}}$
GAPDH	$\dfrac{\mathrm{V_{8f}} \cdot \dfrac{\mathrm{GAP \cdot NAD}}{\mathrm{K \cdot K_{8GAP} \cdot K_{8NAD}}} - \mathrm{V_{8r}} \cdot \mathrm{BPG} \cdot \dfrac{\mathrm{NADH}}{\mathrm{K_{8eq}K_{8GAP}K_{8NAD}}}}{\left(1 + \dfrac{\mathrm{GAP}}{\mathrm{K \cdot K_{8GAP}}} + \dfrac{\mathrm{BPG}}{\mathrm{K_{8BPG}}}\right) \cdot \left(1 + \dfrac{\mathrm{NAD}}{\mathrm{K_{8NAD}}} + \dfrac{\mathrm{NADH}}{\mathrm{K_{8NADH}}}\right)}$
lpPEP	$k_{9f} \cdot \mathrm{BPG} \cdot \mathrm{ADP} - k_{9r} \cdot \mathrm{PEP} \cdot \mathrm{ATP}$
PK	$\dfrac{V_{10m} \cdot \mathrm{ADP} \cdot \mathrm{PEP}}{(K_{10PEP} + \mathrm{PEP}) \cdot (K_{10ADP} + \mathrm{ADP})}$
PDC	$\dfrac{V_{11m} \cdot \mathrm{Pyr}}{K_{11} + \mathrm{Pyr}}$
Glyc	$\dfrac{V_{15m} \cdot \mathrm{DHAP}}{K_{15DHAP} \cdot \left(1 + \frac{K_{15INADH}}{\mathrm{NADH}} \cdot \left(1 + \frac{\mathrm{NAD}}{K_{15INAD}}\right)\right) + \mathrm{DHAP} \cdot \left(1 + \frac{K_{15NADH}}{\mathrm{NADH}} \cdot \left(1 + \frac{\mathrm{NAD}}{K_{15INAD}}\right)\right)}$

Table 3: Kinetic laws in original model

Reaction	Modified Kinetic Law in Reduced Model
PFK	$\dfrac{V_{5m}\cdot\left(\frac{V_{4f}}{V_{4r}}\cdot K_{4eq}\cdot \mathrm{G6P}\right)^2}{K_5\cdot\left(1+\kappa_5\cdot\left(\frac{\mathrm{ATP}}{\mathrm{AMP}}\right)^2\right)+\left(\frac{V_{4f}}{V_{4r}}\cdot K_{4eq}\cdot \mathrm{G6P}\right)^2}$
GAPDH	$\dfrac{\mathrm{V_{8f}}\cdot\frac{\mathrm{trioseP\cdot NAD}}{\mathrm{K\cdot K_{8GAP}\cdot K_{8NAD}}}-\mathrm{V_{8r}}\cdot\frac{\mathrm{BPG\_PEP\cdot k_{9r}\cdot ATP}}{\mathrm{k_{9f}\cdot ADP+k_{9r}\cdot ATP}}\cdot\frac{\mathrm{NADH}}{\mathrm{K_{8eq}K_{8GAP}K_{8NAD}}}}{\left(1+\frac{\mathrm{trioseP}}{\mathrm{K\cdot K_{8GAP}}}+\frac{\mathrm{BPG\_PEP\cdot k_{9r}\cdot ATP}}{\mathrm{k_{9f}\cdot ADP+k_{9r}\cdot ATP}}\cdot\frac{1}{\mathrm{K_{8BPG}}}\right)\cdot\left(1+\frac{\mathrm{NAD}}{\mathrm{K_{8NAD}}}+\frac{\mathrm{NADH}}{\mathrm{K_{8NADH}}}\right)}$
PDC	$\mathrm{V_{10m}}\cdot\dfrac{\frac{\mathrm{BPG\_PEP\cdot k_{9f}\cdot ADP}}{\mathrm{k_{9f}\cdot ADP+k_{9r}\cdot ATP}}\cdot\mathrm{ADP}}{\left(\mathrm{K_{10PEP}}+\frac{\mathrm{BPG\_PEP\cdot k_{9f}\cdot ADP}}{\mathrm{k_{9f}\cdot ADP+k_{9r}\cdot ATP}}\right)\cdot(\mathrm{K_{10ADP}}+\mathrm{ADP})}$
Glyc	$\dfrac{V_{15m}\cdot\frac{\mathrm{trioseP}}{K_{7eq}\cdot K}}{K_{15DHAP}\cdot\left(1+\frac{K_{15INADH}}{\mathrm{NADH}}\right)\cdot\left(1+\frac{\mathrm{NAD}}{K_{15INAD}}\right)+\frac{\mathrm{trioseP}}{K_{7eq}\cdot K}\cdot\left(1+\frac{K_{15NADH}}{\mathrm{NADH}}\cdot\left(1+\frac{NAD}{K_{15INAD}}\right)\right)}$

Table 4: Modified kinetic functions in 5–reaction reduced model

Kinetic Parameter (Reaction)	k_{9f} (lpPEP)	k_{9r} (lpPEP)	$K_{4eq}(PGI)$	K_{6eq} (ALD)	K_{7eq} (TIM)	$V_{10m}(PK)$	$GAP_{st}(ALD)$
Original value	443866	1528.62	0.13	0.081	0.055	343.096	0.12
Values in reduced model	443866	1528.62	0.10439	0.0309765	0.055	343.096	0.255

Table 5: Modified kinetic parameters in 5–reaction reduced model
